# Stalling the Enemy: Targeting Nsp13 for Next-Generation SARS-CoV-2 Antivirals

**DOI:** 10.3390/ijms27062587

**Published:** 2026-03-11

**Authors:** Jose M. Castro, Ryan L. Slack, Yee T. Ong, Huanchun Zhang, Levi B. Gifford, Valentine V. Courouble, Riley M. Aiken, Vishal Shankar, Timothy R. O’Leary, Patrick R. Griffin, Shuiyun Lan, Yuhong Du, Haian Fu, Stefan G. Sarafianos

**Affiliations:** 1 Center for ViroScience and Cure, Laboratory of Biochemical Pharmacology, Department of Pediatrics, Emory University School of Medicine, Atlanta, GA 30322, USA; jose.castro@emory.edu (J.M.C.); ryan.slack@emory.edu (R.L.S.); shuiyun.lan@emory.edu (S.L.); 2 Children’s Healthcare of Atlanta, Atlanta, GA 30329, USA; 3 The Herbert Wertheim Scripps Institute for Biomedical Innovation and Technology, University of Florida Health, Jupiter, FL 33458, USApgriffin2@ufl.edu (P.R.G.); 4 Department of Pharmacology and Chemical Biology, Emory University School of Medicine, Atlanta, GA 30322, USA; dyuhong@emory.edu (Y.D.); hfu@emory.edu (H.F.); 5 Emory Chemical Biology Discovery Center (ECBDC), Emory University, Atlanta, GA 30322, USA

**Keywords:** Nsp13, helicase, SARS-CoV-2, HDX, HTS, antiviral, replicon

## Abstract

The SARS-CoV-2 public health challenges have highlighted the urgent need for coronavirus-targeting life-saving therapeutics. Given the emergence of drug-resistant strains, the development of antivirals against viral proteins beyond the commonly targeted main protease or RNA-dependent RNA polymerase is critical. The SARS-CoV-2 nonstructural protein 13 (nsp13) is a highly conserved RNA helicase and an essential component of the viral replication–transcription complex (RTC). It unwinds double-stranded RNA to facilitate viral transcription and replication, making it a strong target for drug development. To identify nsp13 inhibitors, we used an ultra-high-throughput nucleic acid unwinding assay to screen a library of FDA-approved drugs and bioactive compounds. We identified forty inhibitors with IC_50_ values ranging from 1.4 to 10 μM. Ten were further selected for biochemical and biophysical characterization. Four of these are bound to nsp13 without interacting with the nucleic acid substrate and without inhibiting the ATPase activity of nsp13. Hydrogen–deuterium exchange coupled with Mass Spectrometry (HDX-MS) studies show compound binding causes differential exchange in two regions of nsp13. Furthermore, these compounds have antiviral activity against infectious SARS-CoV-2 in multiple cell lines, with cytotoxicity affecting, in some cases, the apparent antiviral effect. Future optimization efforts could help develop therapeutics against SARS-CoV-2 and other potential coronavirus threats.

## 1. Introduction

In the past two decades, there have been three significant outbreaks of coronaviruses. In 2002, an outbreak of Severe Acute Respiratory Syndrome Coronavirus (SARS-CoV) resulted in 8096 infected cases with a mortality rate of 9.6% [1]. Ten years later, the Middle Eastern Respiratory Syndrome Coronavirus (MERS-CoV) outbreak had a mortality rate of 35.5% among 2254 reported cases [2,3]. The latest is SARS-CoV-2, the causative agent of COVID-19, with more than 776 million cases worldwide and a fatality rate of 1% [4]. Currently approved antivirals have shown a decrease in antiviral efficacy against new variants [5,6,7,8]. Furthermore, some adverse effects and precautions for use have been documented [9,10].

Helicases are NTP-dependent motor proteins that play crucial roles in various cellular and viral nucleic acid processes. They participate in fundamental functions, such as DNA replication, recombination and repair, transcription, translation, and RNA processing [11,12,13,14,15]. All known positive-sense RNA viruses with genomes longer than five kilobases encode a helicase, suggesting they play a role in the replication efficiency of the viral genome [16,17]. An example is viruses within the *Coronaviridae* family, which are characterized by their large positive-sense single-stranded RNA genome of lengths between 26 and 32 kilobases [18,19]. The replication process of coronaviruses involves the synthesis of complementary negative-sense RNA strands, which serve as templates for producing new positive-sense RNA genomes and for discontinuous transcription to generate the necessary subgenomic RNAs needed for the structural and accessory proteins [20,21]. Helicases are responsible for unwinding the double-stranded RNA intermediates during replication, allowing the viral RNA-dependent RNA polymerase to access the templates and synthesize new RNA strands. Thus, helicases are vital for efficient coronavirus replication [22,23,24]. Investigations into regulating helicase activity and identifying potential inhibitors or modulators of coronaviral helicase function could lay the foundation for developing novel antiviral therapies. Since SARS-CoV-2 helicase, nsp13, shares 99.8% and 72% sequence identity with SARS-CoV-1 and MERS-CoV [25,26,27], respectively, inhibitors against one coronavirus helicase may also have a broad antiviral effect against future coronavirus-based epidemics.

We have applied a series of assays to discover and characterize potential inhibitors of SARS-CoV-2 nsp13. We designed an ultra-high throughput screen (uHTS) to assess nsp13 enzymatic activity and used the assay to test a panel of FDA-approved drugs for potential repurposing strategies. We determined the half-maximal inhibitory concentration (IC_50_) values of 74 nsp13 hits. We selected ten hits for further characterization and assessed binding to nsp13, nucleic acid substrates, and the effect on viral ATPase function. Based on these experiments, we tested four compounds for antiviral activity in replicon-based and infectious SARS-CoV-2 assays and demonstrated virus inhibition without cytotoxicity at bioactive concentrations. Collectively, this work validates SARS-CoV-2 nsp13 as a viable antiviral target and identifies a couple of hits that could be used as initial scaffolds for future structure-activity relationship (SAR) studies.

## 2. Results

### 2.1. FRET-Based Unwinding Assay to Measure SARS-CoV-2 nsp13 Activity

**Figure 1:**
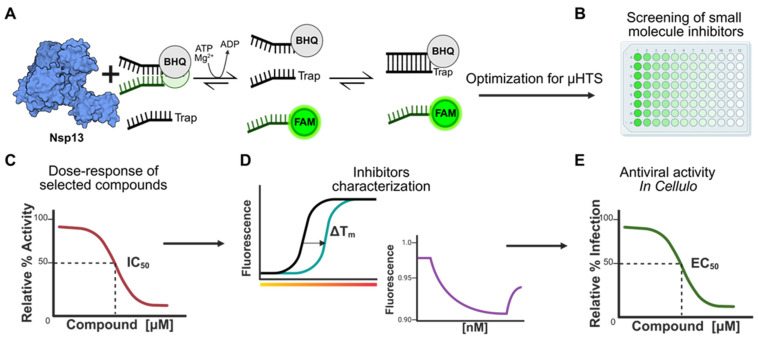
Pipeline for the identification and characterization of potential inhibitors of nsp13 helicase activity. (**A**) Schematic representation of FRET-based nsp13 helicase activity assay. Helicase activity liberates the fluorescently labeled RNA (6-FAM) from the RNA strand containing a fluorescence quencher (BHQ), thereby increasing fluorescence intensity. (**B**) This assay was optimized for ultra-high throughput screening to test chemical libraries for molecules that inhibit nsp13 activity. If a compound inhibits nsp13 unwinding activity, little to no fluorescence is expected. (**C**) Several inhibitory molecules were identified with IC_50_ values ≤ 10 μM. (**D**) Inhibitors were subjected to thermal shift assays (TSA) and microscale thermophoresis assays (MST) for further characterization of their inhibitory properties. (**E**) Inhibitors of nsp13 helicase enzymatic activity were further tested for antiviral activity in cell-based assays using infectious SARS-CoV-2 and SARS-CoV-2 replicon-based systems. Figure created in BioRender. Goins, S. (2026) https://BioRender.com/08k9d3j.

Previously, we developed a Förster Resonance Energy Transfer (FRET)-based assay to quantitatively measure the nucleic acid unwinding activity of SARS-CoV-1 nsp13 (Figure 1A) [28,29,30]. We verified that this assay could also assess the nucleic unwinding activity of SARS-CoV-2 nsp13 (Figure S1A,B). Optimization of conditions resulted in a robust assay as indicated by a Z’ score of 0.62 and a signal-to-background ratio of 11 in 384-well format (Figure S1C,D, plate 2). To identify small molecules that inhibit the helicase activity of recombinant nsp13, we miniaturized the assay to maximize the possible chemical space that can be efficiently screened, given a finite number of resources [31,32,33]. With this in mind, we optimized the SARS-CoV-2 nsp13 activity assay for utilization in 1536-well plate format (Figure S1E–H).

### 2.2. Screening for Small Molecule Inhibitors of nsp13

To screen for inhibitors of the nsp13 nucleic acid unwinding activity, we used the Emory Enriched Bioactive Library (EEBL), which consists of a collection of 2036 diverse small molecules with validated biological and pharmacological activities, including 1018 FDA-approved compounds [34]. We identified 74 compounds that showed more than 40% inhibition of unwinding activity in the presence of 20 µM compound compared to the vehicle control (Table S1) [35,36,37,38,39,40,41,42,43,44,45,46,47,48,49,50,51,52,53,54,55,56,57,58,59,60,61,62,63,64,65,66,67,68,69,70,71,72,73,74,75,76,77,78,79,80,81,82,83,84,85,86,87,88,89,90,91,92,93,94,95,96,97,98,99,100,101,102,103,104,105,106,107,108,109,110,111,112,113,114,115,116,117,118,119,120,121,122,123,124,125,126,127,128,129,130,131,132,133,134,135,136,137,138,139,140,141,142,143,144,145,146,147,148,149,150,151]. We performed dose-response experiments to determine the IC_50_ values for nucleic acid unwinding, which ranged from 1.4 μM to >20 µM.

### 2.3. Validation of Selected Hits from the FRET Screening Assay

**Figure 2:**
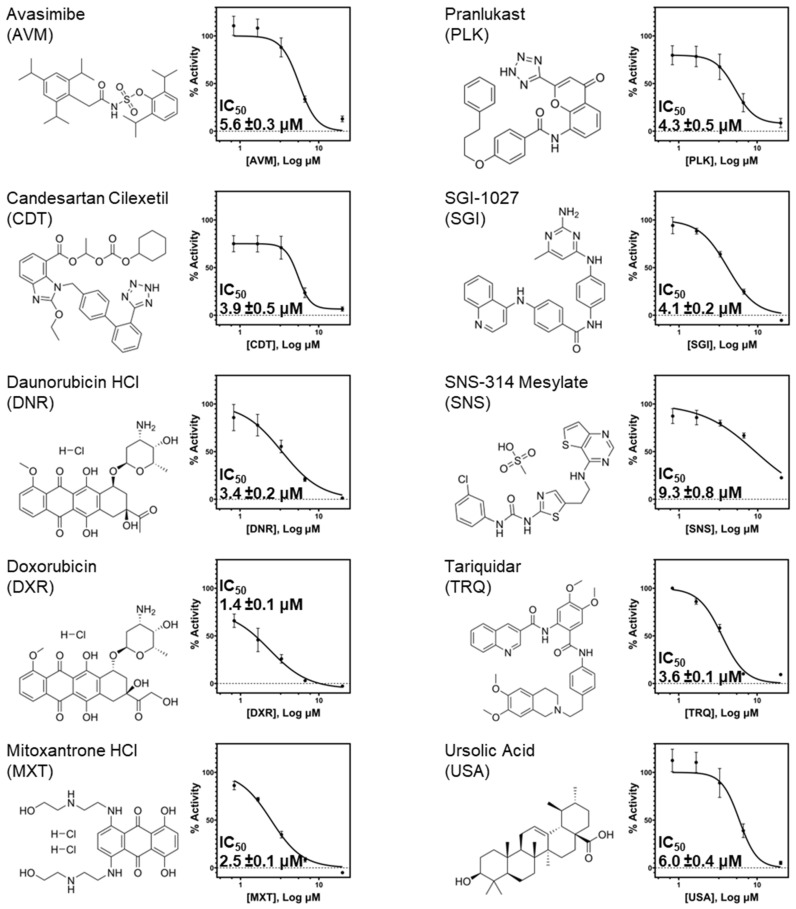
Identification of nsp13 helicase activity inhibitors through uHTS. Ten inhibitors of nsp13 helicase activity were selected for further characterization based on potency and diversity of their reported cellular targets. Hits were tested across a range of concentrations up to 20 μM in quadruplicates. Fitting of dose-response curves to calculate IC_50_ values was performed in GraphPad Prism 10.0.

From these results, we selected ten compounds for further characterization based on their structural and chemical diversity, antiviral potency, and their potential to inhibit by diverse mechanisms of action (Figure 2). These initial hits were validated using an orthogonal native-PAGE helicase assay to confirm their inhibitory effect on nsp13 (Figure S2). Qualitatively, we used this technique to study the enzymatic activity of nsp13 directly by separating the enzymatic substrate and products via polyacrylamide gel electrophoresis under non-denaturing conditions. All tested compounds abrogated the nsp13 nucleic acid unwinding activity in this assay, with AVM, CDT, PLK, and USA showing the most inhibition, followed by SGI, SNS, TRQ, MXT, DXR, and DNR in that order.

### 2.4. Effect of Inhibitors on ATPase Activity of nsp13

**Figure 3:**
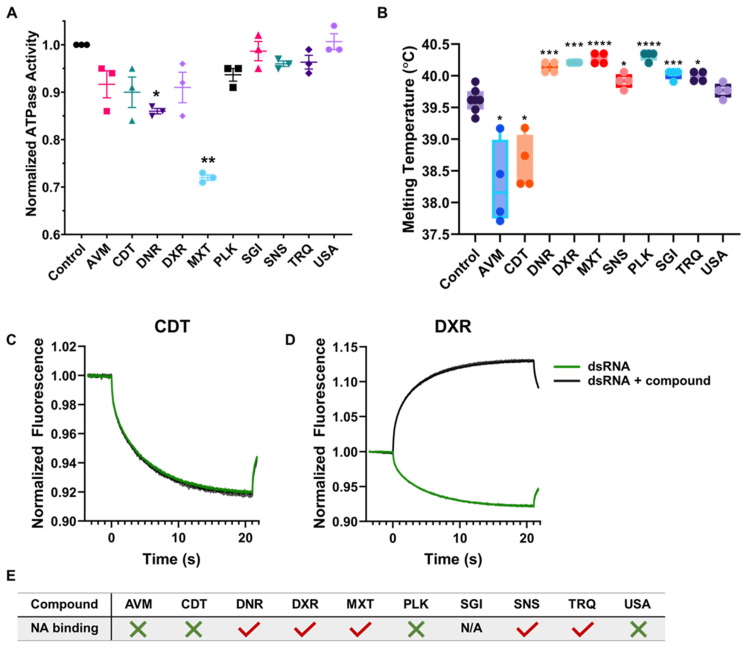
Biochemical and Biophysical characterization of nsp13 helicase activity inhibitors. (**A**) Effect of helicase activity inhibitors on nsp13 ATPase activity. In total, 50 nM nsp13 was exposed to 10 μM of ATP and 20 μM inhibitors (2% DMSO control). Experiments were conducted in triplicate (each data point represents a normalized average against control, per experiment). Error bars reflect the standard deviation of three independent experiments. The statistical analysis includes one-way ANOVA with a *p*-value < 0.05. * *p*-value < 0.05, ** *p*-value < 0.01. Graphical representation performed in GraphPad Prism 10.0. (**B**) Identified compounds bind to nsp13, altering its melting temperature. Nsp13 at 1 μM + 0.2% DMSO as a control, or inhibitors tested at 20 μM. Each sample was tested in quadruplicate (each data point represents a T_m_). Error bars reflect the standard deviation of four independent experiments. Welch’s *t*-test was performed with a *p*-value < 0.05. * *p*-value < 0.05, *** *p*-value < 0.001, and **** *p*-value < 0.0001. Graphical representation of data performed in GraphPad Prism 10.0. (**C**,**D**) Examples of nucleic acid binding assessed by Microscale Thermophoresis (MST). (**C**) MST traces of FAM-labeled dsRNA in the absence (black) or presence (green) of CDT, showing a negligible change in diffusion of the dsRNA. (**D**) MST traces of FAM-labeled dsRNA in the absence (black) or presence (green) of DXR, showing a large change in diffusion of the dsRNA, indicating that this inhibitor directly interacts with the nucleic acid. (**E**) Summary of the outcome for each of the inhibitors. Green X represents compounds that interact with nucleic acid, while red checkmark represents compounds that did not. Figure S4 shows individual thermophoretic traces for each compound.

The uHTS was conducted in the presence of 5 mM of ATP, a high concentration designed to reduce the chances of identifying ATPase inhibitors. To ensure the 10 hit compounds do not interfere with ATP hydrolysis and prevent potential off-target effects, we monitored nsp13 ATPase activity in the presence of inhibitors (Figure 3A). Except for MXT and DNR, all compounds showed no significant difference in nsp13 ATPase activity.

### 2.5. Biophysical Characterization of Small-Molecule Inhibitors of nsp13

#### 2.5.1. Testing for Binding of Inhibitors to nsp13

To test the hypothesis that these compounds function by binding to nsp13, we utilized Differential Scanning Fluorometry (DSF). DSF is a medium-throughput technique used to indirectly detect a small molecule binding to proteins qualitatively [152,153,154]. Compounds that bind nsp13 are expected to affect the protein stability, observable as a change in its melting temperature (T_m_, Figure S3). Notably, the extent of difference in nsp13 T_m_ in the presence of ligand from the unliganded nsp13 T_m_ is indirectly related to ligand binding affinity. Due to the intrinsic fluorescence of several compounds, we routinely employed baseline fluorescence subtraction and intensity normalization for these experiments. All compounds, except for USA, affected the T_m_ of nsp13 under the experimental conditions tested. Specifically, AVM and CDT reduced the T_m_ of nsp13, while DNR, DXR, MXT, SNS, PLK, SGI, and TRQ resulted in a minor increase in the T_m_ of nsp13 (Figure 3B). A decrease in T_m_ can be attributed to compounds that, upon binding, destabilize the protein structure, inducing unfolding at lower temperatures compared to unliganded nsp13. On the other hand, ligands that stabilize the protein increase the melting temperature. Affecting protein stability can disrupt nsp13 activity.

#### 2.5.2. Testing for Binding of Inhibitors to dsRNA

We used microscale thermophoresis (MST) to distinguish whether the inhibitory effect of these compounds is due to direct interactions with nsp13 or indirect interactions with the nucleic acid substrate. This method determines the impact of small molecule binding to a protein or nucleic acid by measuring differences in the diffusion rates of the potential complexes due to changes in the protein or nucleic acid hydration shells, charge, or size [155,156]. We tested differences in the thermophoretic traces of fluorescently labeled double-stranded RNA (dsRNA) in the presence and absence of compounds. Figure 3C,D display representative thermophoretic traces for one inhibitor that does not affect the diffusion of the fluorescently labeled dsRNA (CDT) and another set of traces for an inhibitor that has a large effect on the diffusion of fluorescently labeled dsDNA (DXR). Detailed thermophoretic traces for each compound are shown in Figure S4. From the originally selected compounds, only AVM, CDT, PLK, and USA did not interact with dsRNA and were thus selected for further characterization (Figure 3E).

### 2.6. Using HDX-MS to Investigate Conformational Flexibility of nsp13 upon Ligand Binding

**Figure 4:**
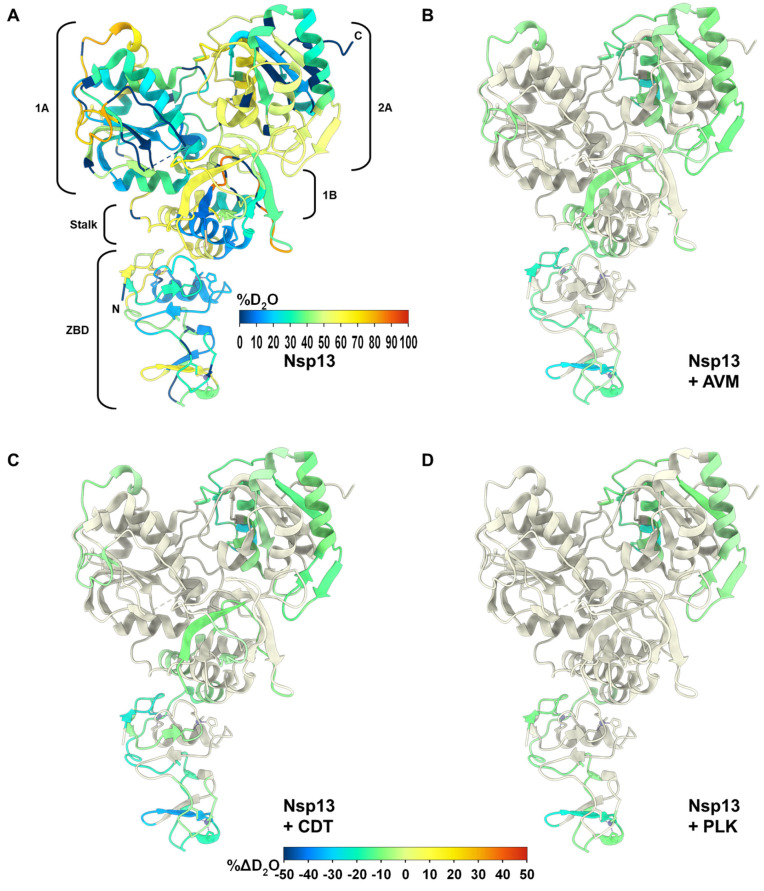
Nsp13 deuterium exchange perturbation plots in the presence of compounds. (**A**) Nsp13 with DMSO (control, representing deuterium uptake). (**B**) Nsp13 with AVM. (**C**) Nsp13 with CDT. (**D**) Nsp13 with PLK. For (**B**–**D**), the colorimetric scale (−50% to 50%) represents the difference in the rate of deuterium exchange between ligand-treated and apo (unliganded) protein. Datum for each amino acid was superimposed on the nsp13 apo structure (PDB: 7NIO). ZBD: Zinc Binding Domain, 1B: beta-barrel 1B domain, 1A: RecA-like domain 1A, and 2A: RecA-like domain 2A. Dashes in the structure represent disordered loops that were unable to be modeled by X-ray crystallography.

We used hydrogen–deuterium exchanged coupled with mass spectrometry (HDX-MS) to explore ligand binding sites and conformational changes to nsp13 in the presence of inhibitors. Amide hydrogens exposed to an aqueous solution are capable of proton exchange. However, residues involved in ligand binding are typically less susceptible to exchange [157,158,159]. Protein regions with higher deuterium exchange are identified through a shift in the isotopic envelope of the corresponding peptide, as detected by mass spectrometry. Data between untreated and treated nsp13 can be compared to identify changes in solvent exposure and hydrogen bonding networks due to the presence of inhibitors. This will help understand nsp13 dynamics and map possible binding sites [160,161,162]. We first exposed nsp13 to a deuterium-based buffer (D_2_O) for a series of predetermined time points to visualize the dynamics of the non-ligated protein. Figure S5A shows the nsp13 deuterium uptake plot, which was overlaid onto the known unliganded nsp13 crystal structure (Figure 4A). We then monitor HDX in the presence of AVM, CDT, PLK, and USA. Differences in deuterium exchange for each compound can be seen in Figure S5B–E which were overlaid as before in Figure 4. USA did not show a deuterium exchange perturbation when compared to the unliganded nsp13. For the other three compounds, Δ% D_2_O can be localized across the different domains in nsp13, with major changes in the RecA-2, and between the zinc-binding and stalk domains. These differences indicate a reduced exchange rate when compared to the untreated nsp13. On an AMP-PNP-bound crystal structure of Nsp13, it was shown that the non-hydrolyzable nucleotide analog interacts between the RecA-1 and RecA-2 domains [163]. In our experiment, none of the reported amino acids interacting with adenylyl-imidodiphosphate (AMP-PNP) from the RecA-1 domain show differences in the rate of deuterium exchange. Similarly, only one residue from the RecA-2 domain that interacts with AMP-PNP shows a difference in deuterium exchange in our experiment. However, a large part of the RecA-2 domain seems to become more rigid in the presence of AVM, CDT, or PLK, as suggested by the reduced deuterium uptake and conformational flexibility.

### 2.7. Antiviral Efficacy of nsp13 Inhibitors in Reporter-Replicon Assays and Stably Expressing Reporter-Replicon Cell Line

**Figure 5:**
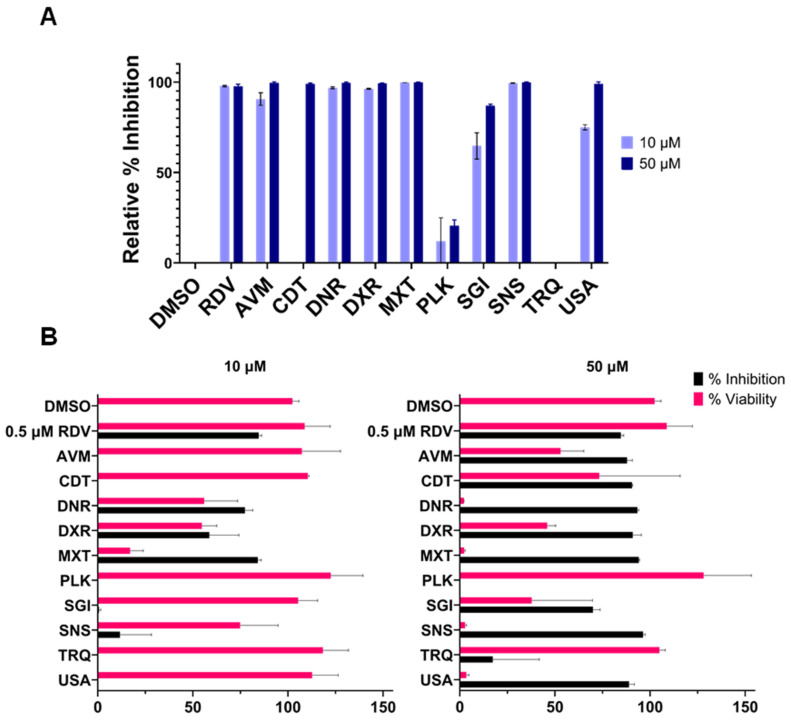
Nsp13 helicase inhibitors tested in a transient transfection or a stable cell line system expressing SARS-CoV-2 subgenomic replicon (SARS-2R). (**A**) HEK293 cells were transfected with SARS-2R expressing NanoLuc Luciferase as a reporter. Transfected cells were treated with inhibitors at 10 or 50 μM. After a 2-day incubation, NanoLuc substrate was added, and luminescence was measured using a Promega luminometer. (**B**) BHK-21 cells stably expressing SARS-2R with a NanoLuc Luciferase reporter were seeded into a plate with inhibitor at 10 or 50 μM (**B**). After a 2-day incubation, NanoLuc luciferase activity was measured as above. For measuring % cell viability, the media was removed, and PrestoBlue was added. The data represent the mean of two independent experiments with two technical replicates each. Remdesivir (RDV) was used as a positive control. For both experiments, graphical representation and statistical analysis were performed in GraphPad Prism 10.0.

After characterizing the selected compounds biochemically and biophysically, we further examined their antiviral efficacy using HEK293 cells transiently expressing a SARS-CoV-2 replicon (SARS-2R) with a luciferase reporter to monitor viral replication [164]. Except for viral entry and egress, our SARS-2R replicon system recapitulates the SARS-CoV-2 replication cycle, with viral transcription levels directly proportional to luciferase activity. Therefore, compounds that inhibit nsp13 helicase activity are expected to reduce luciferase activity. In initial experiments, transfected cells expressing the replicon (as monitored by eGFP expression) were treated with compounds at 10 and 50 µM (Figure 5A). The nucleoside analog Remdesivir (RDV), which targets the SARS-CoV-2 RNA-dependent RNA polymerase, was used as a positive control. At 10 µM AVM, DNR, MXT, and SNS demonstrated more than 50% inhibition. At 10 µM AVM, DNR, DXR, MXT, SGI, SNS, and USA demonstrated more than 50% inhibition. At 50 µM AVM, CDT, DNR, DXR, MXT, SNS, and USA showed >95% inhibition of viral transcription.

While transient transfection of SARS-2R allows for rapid and efficient transcription and expression, we also sought to test the effect of these inhibitors in a stable transfection system, where replicon expression is more sustained and uniform, reducing the variability associated with transient transfection. To this end, we evaluated the antiviral activity of these compounds in our stably transfected BHK-21 SARS-2R cell line. The cells were treated with 10 and 50 µM compounds under the same conditions previously tested in HEK293 cells (Figure 5B). We assessed both inhibition and cytotoxicity at these two concentrations. At 10 µM, DNR, DXR, and MXT showed inhibition levels comparable to those in HEK293 cells transiently transfected with the replicon. At 50 µM, AVM, CDT, DNR, DXR, MXT, SNS, and USA effectively inhibited luciferase activity in the BHK-21 SARS-2R cells. However, at 50 µM, most compounds exhibited different levels of cytotoxicity as assessed by the PrestoBlue assay.

### 2.8. Antiviral Efficacy of Selected Inhibitors of nsp13 Against SARS-CoV-2 Infection

Finally, we assessed the antiviral activity of AVM, CDT, PLK, and USA against SARS-CoV-2 across several cell lines: (1) Vero-E6-ACE2 cells or African green monkey kidney cells, which are highly susceptible to CoV replication due to their lack of type I interferon genes, stably expressing the human Angiotensin-converting 2 [ACE2] receptor that is required for entry of the infectious virus. (2) Huh-7.5 or human hepatoma-derived cells with a mutation in the CARD domain of RIG-I that disrupts interferon response to RNA viruses. (3) Caco-2 or human epithelial colorectal adenocarcinoma cells. (4) Calu-3 or human lung adenocarcinoma cells. All cell lines were exposed to the compounds at various concentrations one hour before infection with ic-SARS-CoV-2/WA1-mNG at 30 IU. Forty-eight hours post-infection, the cells were fixed, and fluorescence was measured to calculate each compound’s half-maximal effective concentration (EC_50_). We further evaluated the cytotoxicity of these compounds using XTT or nuclear staining. Table 1 summarizes the antiviral activity of the four compounds. AVM demonstrated antiviral activity in all five cell lines, with EC_50_ values ranging from 1.7 μM in Caco-2 cells to 9.08 μM in VeroE6 cells. The selectivity index was highest in Caco-2 cells (SI = 7.1) and lowest in Vero-E6 cells (SI = 1.6), indicating variable therapeutic windows depending on the host cell type. CDT exhibited weak or undetectable antiviral activity in most cell lines, with EC_50_ values >25 μM in Vero-E6-ACE2 and Huh-7.5. Modest activity was observed in Caco-2 (EC_50_ = 21.9, SI = 1.9) and Calu-3 cells (EC_50_ = 17.7 μM, SI = 2.8). PLK showed a similar profile as CDT, with EC_50_ values >25 μM in most cell lines. Some antiviral activity was observed in Caco-2 cells (EC_50_ = 20 μM, CC_50_ > 50 μM, SI >2.4), but EC_50_ values exceeded 50 μM in Calu-3 cells, suggesting low efficacy. USA demonstrated a mixed profile. While antiviral activity was evident in Vero-E6-ACE2 (EC_50_ = 20.9 μM), Caco-2 (EC_50_ = 4.6 μM), and Calu-3 (EC_50_ = 9.7 μM), cytotoxicity was more prominent in Vero-E6-ACE2 with CC_50_ values of 12.5 μM.

## 3. Discussion

In this study, we used in vitro uHTS, orthogonal validation assays, and mechanistic characterization of selected initial hits to identify SARS-CoV-2 antivirals that act by inhibiting the helicase activity of nsp13. This strategy led to the identification of four compound leads for potential future ligand-based drug design and structure-activity relationship (SAR) studies.

Avasimibe (AVM) was developed as an Acyl-coenzyme A:cholesterol acyltransferase (ACAT) inhibitor for the treatment of atherosclerosis [165]. Inhibition of the nsp13 helicase activity by AVM is consistent with a previous report [166]. Here, we also demonstrate that AVM blocks SARS-CoV-2 replication in cell-based assays using either replicon-based systems (transfection or stable cell lines) or fully infectious SARS-CoV-2. It is described as an inhibitor of nsp3 and nsp13 as identified by in vitro high-throughput assays [166,167]. As it disrupts the association of SARS-CoV-2 pseudoviruses with their cognate ACE-2 receptors, it has been proposed to perturb viral attachment and entry [168]. However, we show here that AVM also inhibits our replicon-based system that does not involve cellular entry. This suggests that AVM has a multifaceted mechanism of action. This is also consistent with previously published data showing a reduction in viral RNA and replication foci upon infection [168]. Comparison of the inhibition of infectious SARS-CoV-2 with the inhibition of the replicon system (Figure 5) suggests that the inhibition of nsp13 is the dominant mechanism. It has also been proposed that AVM enhances SARS-CoV-2-specific T cell responses [168]. However, our study is the first to describe a specific mechanism by which AVM blocks the helicase activity of SARS-CoV-2, without binding the viral nucleic acid and without affecting the ATPase activity of nsp13. It also provides experimental evidence that AVM binding induces structural perturbation in two regions of nsp13.

Regarding the antiviral mechanism of Candesartan Cilexetil (CDT), comparison of the data using a fully infectious virus (Table 1) versus the replicon system (Figure 5) or purified nsp13 enzyme suggests that the antiviral effect of CDT primarily stems from its inhibition of the nsp13 helicase activity. However, CDT may have an additional benefit that complements its antiviral activity, and it is used as an angiotensin II receptor blocker (ARB) to treat hypertension [169]. Notably, hypertension and cardiovascular disease are associated with the severity of COVID-19 illness [170,171]. The SARS-CoV-2 Spike protein binds to host ACE-2 receptors to facilitate viral entry. The use of ARBs is known to increase ACE-2 expression, initially raising concerns about increased risk for millions of patients with chronic cardiovascular disease or hypertension [172,173]. Surprisingly, several studies have demonstrated that enhanced ACE-2 expression protects the lungs from acute respiratory distress syndrome (ARDS) [174,175,176]. Hence, CDT would be expected to confer an added benefit in addition to its antiviral activity. Indeed, a prospective clinical trial showed that CDT treatment in non-obese COVID-19 patients resulted in reduced length of hospital stays, time to improvement on chest X-ray tests, and length of time to produce a negative COVID-19 nasal swab [177].

Pranlukast (PLK) is a cysteinyl leukotriene (CysLT1) receptor antagonist developed for the treatment of chronic asthma [178,179,180]. Leukotrienes are signaling lipid molecules produced in response to stimuli, such as allergens, that subsequently bind extracellular leukotriene receptors and induce bronchoconstriction and inflammation, which is suppressed by CysLT1 antagonist binding. While we found that PLK inhibits the enzymatic activity of nsp13 in vitro, it did not exhibit antiviral activity in cells. This lack of antiviral efficacy may have been anticipated since PLK primarily targets extracellular receptors, which may restrict its ability to traverse the cell membrane and thus affect viral replication inside cells. Moreover, to our knowledge, there is no direct evidence that PLK enters target cells. As such, it would be unable to exert an antiviral effect against SARS-CoV-2. Future experiments with analogs known to traverse the plasma membrane may result in compounds with antiviral properties. Nevertheless, the potential use of CysLT1 antagonists as a COVID-19 therapy has been noted by others for their efficacy in reducing prognostic indicators of ARDS [181,182]. Notably, another CysLT1 antagonist, Montelukast sodium, also inhibited nsp13 activity in our initial screen for nsp13 inhibitors (Table S1). These CysLT1 antagonists may provide molecular scaffolds for the design of nsp13 inhibitors with improved cellular permeability.

Ursolic acid (USA) is a pentacyclic triterpenoid found in various berries, coffee, apples, herbs, olives, and some spices [183]. It has been associated with multiple therapeutic benefits, including anti-inflammatory, antioxidant, anti-carcinogenic, and anti-apoptotic properties [184,185,186,187]. Additionally, USA has demonstrated antiviral effects against human immunodeficiency virus type 1 (HIV-1) and Hepatitis C virus (HCV) [188,189]. This has led to speculation that USA might help alleviate some COVID-19 symptoms [190,191]. In our study, USA inhibited the in vitro activity of SARS-CoV-2 nsp13. However, HDX-MS experiments did not show USA interacting with nsp13, although we cannot exclude the possibility that it may bind the nsp13-RNA or RTC-nsp13 complexes. Furthermore, USA showed modest antiviral activity compared to cytotoxicity in cell-based assays (Figure 5, Table 1). Other studies have shown that the therapeutic benefits of USA are limited by the compound’s poor bioavailability and absorption [192,193,194]. This may partially explain the limited antiviral efficacy we observed in cell-based assays.

HDX-MS data for AVM, CDT, and PLK reveal significant perturbation differences in the RecA-like domains and the Zinc Binding Domain (ZBD) of nsp13. The RecA-like domains are critical for the ATPase activity of nsp13. These domains, along with the 1B domain, form a tunnel through which the viral RNA substrate is proposed to pass, facilitating the replication and transcription of viral RNA as well as the proposed backtracking activity that nsp13 is said to exert [195,196]. The reported ATPase activity assays indicate that none of the three compounds inhibit the conversion of ATP to ADP, suggesting their antiviral properties may stem from disrupting nsp13-RNA interactions within these domains. The ZBD also exhibits reduced hydrogen-deuterium exchange in the presence of these compounds, indicative of an increased structural rigidity. The ZBD is known to mediate interactions between nsp13 and other RTC components, such as the RNA-dependent RNA polymerase (nsp12) and nsp8 [196,197]. AVM, CDT, and PLK may stall nsp13 and hinder its ability to bind the RTC, leading to suppression of helicase function and unwinding of viral RNA. This mechanism could represent a novel antiviral strategy targeting the essential role of nsp13 in viral replication.

Targeting the highly conserved beta-coronavirus nsp13 would allow the development of a novel set of antivirals that could complement available therapeutics against SARS-CoV-2 and other potential coronavirus threats. In this study, we identified, biochemically validated, and characterized compounds that inhibited nsp13 in vitro and in cellular studies. The selectivity indices of some of the compounds suggest that part of the observed antiviral activity could be attributed to compound-associated cytotoxicity. Although the selected compounds have prior clinical validation, they require optimization before consideration for SARS-CoV-2 treatment. These initial hits demonstrate that nsp13 is a viable antiviral target and can be considered as scaffolds for further SAR studies toward the development of future treatments for SARS-CoV-2 infection.

## 4. Materials and Methods

### 4.1. Materials

Hit compounds from the screen, Avasimibe, Candesartan Cilexetil, Daunorubicin HCl, Doxorubicin, Mitoxantrone HCl, Pranlukast, SGI-1027, SNS-314 Mesylate, Tariquidar, and Ursolic Acid, were purchased from MedChemExpress (Monmouth Junction, NJ, USA).

COVID-SARS2 NSP13 plasmid was obtained through Addgene (plasmid # 159614) [163].

HEK293 cells were obtained from the American Type Culture Collection (ATCC, Manassas, VA, USA) and are a human embryonic kidney-derived cell line. BHK-21 (ATCC) are derived from baby hamster kidney and may have defects in IFN production and response to IFN [198,199]. Vero-E6-ACE2 (ATCC) are derived from African green monkey kidney cells and lack the genes encoding type I interferons. Huh-7.5 (provided by Charles Rice) is derived from human hepatoma cells. Caco-2 cells (ATCC) are a heterogeneous population derived from human colorectal adenocarcinoma, and Calu-3 cells (ATCC) are a heterogeneous population derived from human lung adenocarcinoma tissue.

SARS-CoV-2 replicon based on parental Washington (WA-1) strain was previously generated in our lab (SARS-2R) [164]. An infectious clone of SARS-CoV-2 WA-1 containing the mNeonGreen reporter (ic-SARS-CoV-2/WA1-mNG) was obtained from the World Reference Center of Emerging Viruses and Arboviruses (WRCEVA) at the University of Texas Medical Branch [200].

### 4.2. Protein Expression and Purification

The His-nsp13 plasmid was transformed into competent *E. coli* NiCo21 (DE3) cells (NEB). Cells were grown in 4 L of terrific broth at 37 °C and raised to an OD_600_ of 0.7 in the presence of kanamycin. The cultures were cooled to 16 °C and induced with 0.1 mM IPTG for 16 h. One-liter bacterial pellets were resuspended to a total volume of 50 mL lysis buffer (50 mM HEPES, 500 mM NaCl, 10 mM imidazole, 0.5 mM TCEP, 5% glycerol, pH 7.5) and lysozyme, DNase, and RNase were added to degrade the cell wall and remove excess DNA and RNA. Resuspended cells were then sonicated for 12 min (10 s on and 5 s off at 80% power). The lysate was clarified by centrifugation at 20,000× *g* RPM, 4 °C for 30 min. The resulting supernatant was transferred to a 50 mL conical tube containing 1 mL of His-Select Nickel Affinity Gel (Sigma-Aldrich, St. Louis, MO, USA) and incubated for 1 h at 4 °C on a gel rocker. The affinity beads were washed with 80 mL lysis buffer and eluted with 15 mL of elution buffer (50 mM HEPES, 500 mM NaCl, 300 mM imidazole, 0.5 mM TCEP, 5% glycerol, and pH 7.5). The eluate was loaded onto a Mono-S ion exchange column (pre-equilibrated with elution buffer). The Mono-S column was washed with 20 mL elution buffer, and the residual protein was eluted with 15 mL high salt buffer (50 mM HEPES, 1 M NaCl, 0.5 mM TCEP, 5% glycerol, and pH 7.5). The Mono-S flow-through was concentrated and loaded onto a Superdex 200 Increase 10/300 column (equilibrated with 50 mM HEPES, 500 mM NaCl, 0.5 mM TCEP, 5% glycerol, and pH 7.5). The fractions containing nsp13 were concentrated, flash-frozen, and stored at −80 °C.

### 4.3. Ultra-High Throughput Screen (uHTS)

The uHTS was performed using a fluorescence-based assay in black 1536-well plates (Corning Costar, #3724, Corning, NY, USA) with a total volume of 5 μL in each well. Briefly, 4 μL of mixture containing 5 nM nsp13, 50 nM annealed 6-FAM/BHQ DNA dsDNA and 2 μM BHQ Trap was dispensed into a 1536-well plate using Multidrop Combi (Fluorescent strand: 6FAM/5′-CCAGGCTCAGATACGACCACCACT-3′, Quencher strand: 5′-TCACCACCACGTATCTGAGCCTGG-3′/BHQ, and Trap: 5′-TCACCACCACGTATCTGAGCCTG G-3′). In total, 0.1 μL of compounds was added into the wells using a pin tool integrated with the Beckman NX liquid handler (Beckman Coulter, Brea, CA, USA). The final concentration of compounds is 20 μM. After incubating at room temperature for 30 min, 1 μL of ATP (5 mM final) was dispensed into the wells. Fluorescence intensity (FI) was measured using a BMG LABTECH PHERAstar FSX plate reader (BMG Labtech, Ortenberg, Germany) with Ex. 480 nm and Em. 520 nm for 6-FAM.

### 4.4. Evaluation of IC_50_s for Selected Compounds Identified by uHTS

From the library tested, 74 compounds that show >40% nsp13 activity inhibition at 20 μM were selected for dose-response studies. Compounds were tested at a range from 20–0.83 μM in quadruplicate using the same conditions as the uHTS screening. The IC_50_s were analyzed using GraphPad Prism 10 (GraphPad Software, LLC, Boston, MA, USA).

### 4.5. Native-PAGE Helicase Assays

Reaction mixtures were comprised of 30 nM nsp13, 50 nM annealed DNA, 2 mM ATP, 1 µM trap DNA, and reaction buffer (20 mM HEPES, 20 mM NaCl, 0.1 mM TCEP, 5 mM MgCl_2_, 0.05% BSA, 5% glycerol, and pH 7.5). Nsp13 was preincubated with the reaction buffer and annealed DNA to allow for interaction. ATP was added last at a concentration of 2 mM. The reaction was incubated at 30 °C for ten minutes and quenched with an equal volume of quenching buffer (100 mM EDTA, 20% glycerol, and 0.5% bromophenol blue). The quenched reaction mixture was transferred to a 16% tris-glycine native-PAGE gel at room temperature and run at a constant voltage of 130 V. Imaging was performed on a ChemiDoc imaging system (Bio-Rad, Hercules, CA, USA) using the SYBR-safe blue light setting to measure FAM-oligo electrophoretic shifts. For DNA gels, the same DNA oligos were used as before (without quencher). For RNA gels, the RNA equivalent of the DNA sequence was used.

### 4.6. Nsp13 ATPase Activity Assay

ADP-GLO assay (Promega Corp., Madison, WI, USA) was used. Purified nsp13 was diluted to 1 µM in reaction buffer (50 mM Tris, 100 mM NaCl, 0.5 mM TCEP, and 20 mM MgCl_2_ at pH 7.5) for a final concentration of 50 nM in the reaction. Inhibitors of interest were added to diluted nsp13 at a concentration of 20 µM and incubated at room temperature for 20 min. Duplex DNA previously annealed was added to the reactions at a concentration of 200 nM, followed by ATP at a final concentration of 10 µM. The reaction was incubated at 30 °C for 10 min. The 25 μL L sample reactions were then transferred to a 96-well plate, followed by the addition of 25 µL of ADP-GLO reagent. The plate was incubated for 40 min at room temperature. Finally, 50 μL of the kinase detection reagent was added to each well, and the plate was incubated at room temperature for 30 min. Luminescence was measured with the Promega GloMax navigator plate reader. Hydrolyzed ATP was calculated based on the standard curve.

### 4.7. DSF/TSA

Purified nsp13 was diluted to 1 µM in reaction buffer (20 mM HEPES, 20 mM NaCl, 0.2 mM TCEP, 5 mM MgCl_2_, and 5% glycerol at pH 7.5). Inhibitors of interest were added to the nsp13 solution at a concentration of 20 µM. Reactions were mixed with the final 1X GloMelt (biotium) stain. Reactions were set on a PCR plate and heated from 25 to 95 °C with a heating rate of 0.2 °C every 10 s in the QuantStudio 3 PCR system (Thermo Fisher Scientific, Waltham, MA, USA). Fluorescence intensity will be measured (excitation: 495 nm and emission: 520–590 nm). The data were analyzed with the Protein Thermal Shift Software version 1.4 (Thermo Fisher Scientific).

### 4.8. Microscale Thermophoresis

Duplex RNA was annealed by mixing a 1:1.2 ratio of FAM-oligo–unlabeled-oligo in annealing buffer (30 mM HEPES, 60 mM KOAc, 0.2 mM MgCl_2_, and pH 7.5). The oligos were annealed by heating the solution to 90 °C for 5 min, then incubating the solution at room temperature. The annealed RNA was added to the MST buffer (20 mM HEPES, 20 mM NaCl, 0.1 mM TCEP, 5 mM MgCl_2_, 5% glycerol, and pH 7.5) at a final concentration of 50 nM in the presence or absence of an inhibitor at a final concentration of 20 µM. Samples were transferred to NT.115 series premium capillaries (Nanotemper, Munich, Germany), and MST experiments were performed and analyzed using a Monolith NT.115 (Nanotemper). FAM fluorescence was monitored using the nano-blue channel (excitation 493 nm and emission 521 nm), with an excitation power of 80%, medium IR-laser power, and IR-laser on-time of 20 s.

### 4.9. Hydrogen–Deuterium Exchange (HDX) Detected by Mass Spectrometry (MS)

Peptide Identification: Differential HDX-MS experiments were conducted as previously described with a few modifications [159]. Prior to the HDX-MS experiment, pepsin-digested Nsp13 peptides were identified using tandem MS (MS/MS) with an Orbitrap mass spectrometer (Q Exactive, Thermo Fisher Scientific). Spectra were acquired in data-dependent mode with the top five most abundant ions selected for MS2 analysis per scan event. The MS/MS data files were submitted to Mascot for analysis. Peptides identified with an accuracy of 10 ppm of the theoretical *m*/*z* and an ion score of 20 or greater were included in the final peptide set. HDX-MS analysis: Nsp13 (10 µM concentration, in 20 mM HEPES, 20 mM NaCl, 0.2 mM TCEP, 5 mM MgCl_2_, and pH 7.5) was incubated with 100 µM compound or DMSO (30 min. at 4 °C). Following the incubation, 5 μL of the sample was diluted into 20 μL D_2_O buffer (20 mM HEPES, 20 mM NaCl, 0.2 mM TCEP, 5 mM MgCl_2_, pH 7.5) and incubated for various time points (0, 10, 60, 300, 900, and 3600 s) at 4 °C. The deuterium exchange was then slowed by mixing with 25 μL of cold (4 °C) 0.1 M sodium phosphate monobasic with 50 mM TCEP. Upon injection, quenched samples were passed through a pepsin protease column (1 mm × 2 cm) at 50 μL min^−1^, and the digested peptides were captured on a 1 mm × 1 cm C8 trap column (Agilent) and desalted. Peptides were separated across a 1 mm × 5 cm C18 column (1.9 μL Hypersil Gold, Thermo Fisher Scientific) with a linear gradient of 4–40% CH3CN and 0.3% formic acid, over 5 min. Sample handling, protein digestion, and peptide separation were conducted at 4 °C. The mass spectrometric data were acquired using an Orbitrap mass spectrometer (Q Exactive, Thermo Fisher Scientific). HDX analyses were performed in triplicate from single preparations. The intensity-weighted mean *m*/*z* centroid value of each peptide envelope was calculated and subsequently converted into a percentage of deuterium incorporation. This is accomplished by determining the observed averages of the undeuterated and fully deuterated spectra and using the conventional formula described elsewhere [201]. Statistical significance of the differential HDX data is assessed using an unpaired *t*-test at each time point, a procedure integrated into the HDX Workbench software [202]. Corrections for back-exchange were made on the basis of an estimated 70% deuterium recovery, and accounting for the known 80% deuterium content of the deuterium exchange buffer. Data Rendering: The HDX data from all overlapping peptides were consolidated to individual amino acid values using a residue averaging approach. Briefly, for each residue, the deuterium incorporation values and peptide lengths from all overlapping peptides were assembled. A weighting function was applied in which shorter peptides were weighted more heavily and longer peptides were weighted less. Each of the weighted deuterium incorporation values was then averaged to produce a single value for each amino acid. The initial two residues of each peptide, as well as prolines, were omitted from the calculations. This approach is similar to that previously described [203].

### 4.10. Replicon NanoLuc Luciferase Assays

BHK-21 SARS-CoV-2 replicon-expressing cells, based on the Washington strain (WA-1) [164], plated in 96-well culture plates, were incubated with nsp13 inhibitors at either 10 or 50 μM with a consistent DMSO concentration for all inhibitor concentrations (the highest concentration in each experiment was kept consistent). Inhibitors were incubated with cells for 48 h. After incubation, 50 µL of the culture supernatant was collected and added to a different 96-well plate. To these wells, nano-luciferin (1:50 dilution in NanoLuc buffer) was added at 50 µL per well to a final volume of 100 µL. The plate was immediately analyzed using the Glo-max instrument to obtain the relative light unit (RLU) per treatment.

### 4.11. Infectivity Assay EC_50_

Experiments were performed in 96-well plates. Cells were seeded at 30,000 cells per well a day prior to the experiment. Cells were treated with compounds 1 h prior to infection at varying concentrations, with a consistent DMSO concentration for all inhibitor concentrations (the highest concentration in each experiment was kept consistent). They were infected with 300 IU/well of ic-SARS-CoV-2/WA1-mNG. Cells were fixed 2 dpi with 4% paraformaldehyde for 30 min at room temperature, followed by 1X DPBS wash and incubated with 0.05% Triton X-100 with HOECHST 33342. After nuclei were stained for 30 min, the stain was removed with a 1X DPBS wash prior to imaging GFP-positive cells with Cytation5 (BioTek, Agilent technologies, Santa Clara, CA, USA).

### 4.12. Cell Viability

Cell viability in the presence of hit compounds was tested by PrestoBlue or XTT assays. HEK293 and BHK-21 cells were treated for 48 h with 10 or 50 µM compound. After treatment, cells were exposed to a 1:10 ratio of PrestoBlue to media (Thermo Fisher Scientific), incubated for 10 min, and fluorescence was measured using a BioTek Synergy microplate reader (Agilent Technologies, Santa Clara, CA, USA). Viability was calculated as a percentage compared to the DMSO control.

Vero-E6-ACE2, Huh-7.5, Caco-2, and Calu-3 cells were treated for 48 h with 25 or 50 µM compound. After treatment, cell viability was assessed using an XTT kit (Roche, Basel, Switzerland) according to the manufacturer’s instructions. Cell viability in the presence of the compound was normalized against the cell viability of DMSO-treated cells. Values were plotted in GraphPad Prism and analyzed using the log (inhibitor) versus normalized response—variable slope equation to obtain CC_50_ values.

## Figures and Tables

**Table 1:** Antiviral activity of inhibitors against SARS-CoV-2 infection in different cell lines. EC_50_ and CC_50_ reported in μM concentrations. SI: Selectivity Index (CC_50_/EC_50_). ND cannot be determined. ^a^ Cytotoxicity determined by XTT assay. ^b^ Cytotoxicity was determined based on nuclear staining (Hoechst stain). ^c^ Half-maximal effective concentration determined with SARS-CoV-2 WA-1 strain. All other EC_50_ were obtained using icSARS2/WA1-mNG.

	Vero-E6-ACE2			Huh-7.5			Caco-2			Calu-3		
	EC_50_	CC_50_ ^a^	SI	EC_50_	CC_50_ ^a^	SI	EC_50_	CC_50_ ^b^	SI	EC_50_	CC_50_ ^b^	SI
RDV	2.7 ± 0.3 ^c^	>5	>1.9	0.006 ± 0.002 ^c^	>5	>833.3	0.02 ± 0.004	>20.8	>1040	0.23 ± 0.22	>20.8	>90.4
AVM	9.08	14.2	1.6	5.1 ± 1.5	14.0 ± 2.6	2.7	1.7 ± 0.4	12.1 ± 1.7	7.1	7.8 ± 1.7	28.4 ± 2.6	3.6
CDT	>25	>25	ND	>25	>25	ND	21.9 ± 1.8	42.2 ± 1.2	1.9	17.7 ± 0.1	>50	2.8
PLK	>25	>25	ND	>25	>25	ND	20.9 ± 1.7	>50	>2.4	>50	>50	ND
USA	20.9	12.5	0.6	>25	>25	ND	4.6 ± 0.7	33.6 ± 4.9	7.3	9.7 ± 0.1	36.7 ± 3.4	3.8

## Data Availability

The original contributions presented in this study are included in the article/Supplementary Materials. Further information and requests for resources and reagents should be directed to and will be fulfilled by the corresponding author, Stefan G. Sarafianos (stefanos.sarafianos@emory.edu).

## Supplementary Materials

The supporting information can be downloaded at: https://www.mdpi.com/article/10.3390/ijms27062587/s1.

## Abbreviations

ACAT	Acyl-coenzyme A: cholesterol acyltransferase
ACE-2	Angiotensin-converting enzyme 2
AMP-PNP	Adenylyl-imidodiphosphate
ARB	Angiotensin 2 receptor blocker
ARDS	Acute Respiratory Distress Syndrome
ATP	Adenosine triphosphate
ATPase	Adenosine triphosphatase
AVM	Avasimibe
CDT	Candesartan Cilexetil
COVID-19	Coronavirus Disease of 2019
CysLT1	Cysteinyl Leukotriene 1 receptor
DNR	Daunorubicin HCl
DSF	Differential Scanning Fluorometry
dsRNA	Double-stranded RNA
DXR	Doxorubicin
eGFP	Enhanced Green Fluorescent Protein
FRET	Förster Resonance Energy Transfer
HDX-MS	Hydrogen-Deuterium exchange coupled with Mass Spectrometry
MERS-CoV	Middle Eastern Respiratory Syndrome Coronavirus
MST	MicroScale Thermophoresis
MXT	Mitoxantrone HCl
Nsp13	Nonstructural protein 13
PLK	Pranlukast
RDV	Remdesivir
RecA-2	RecA-like
RTC	Replication Transcription Complex
SAR	Structure-Activity Relationship
SARS-2R	SARS-CoV-2 replicon
SARS-CoV	Severe Acute Respiratory Syndrome Coronavirus
SGI	SGI-1027
SNS	SNS-314 Mesylate
TRQ	Tariquidar
TSA	Therma Shift Assay
uHTS	Ultra-high throughput screening
USA	Ursolic acid
ZBD	Zinc-binding domain
