# Supplementary material for: Stalling the Enemy: Targeting Nsp13 for Next-Generation SARS-CoV-2 Antivirals
Source: Int J Mol Sci. 2026 Mar 11;27(6):2587. doi: 10.3390/ijms27062587 (PMC13027058; doi:10.3390/ijms27062587)
Supplement: Supplementary file 1 [file ijms-27-02587-s001.zip › ijms-4163309-supplementary.pdf]

Supplemental Figures

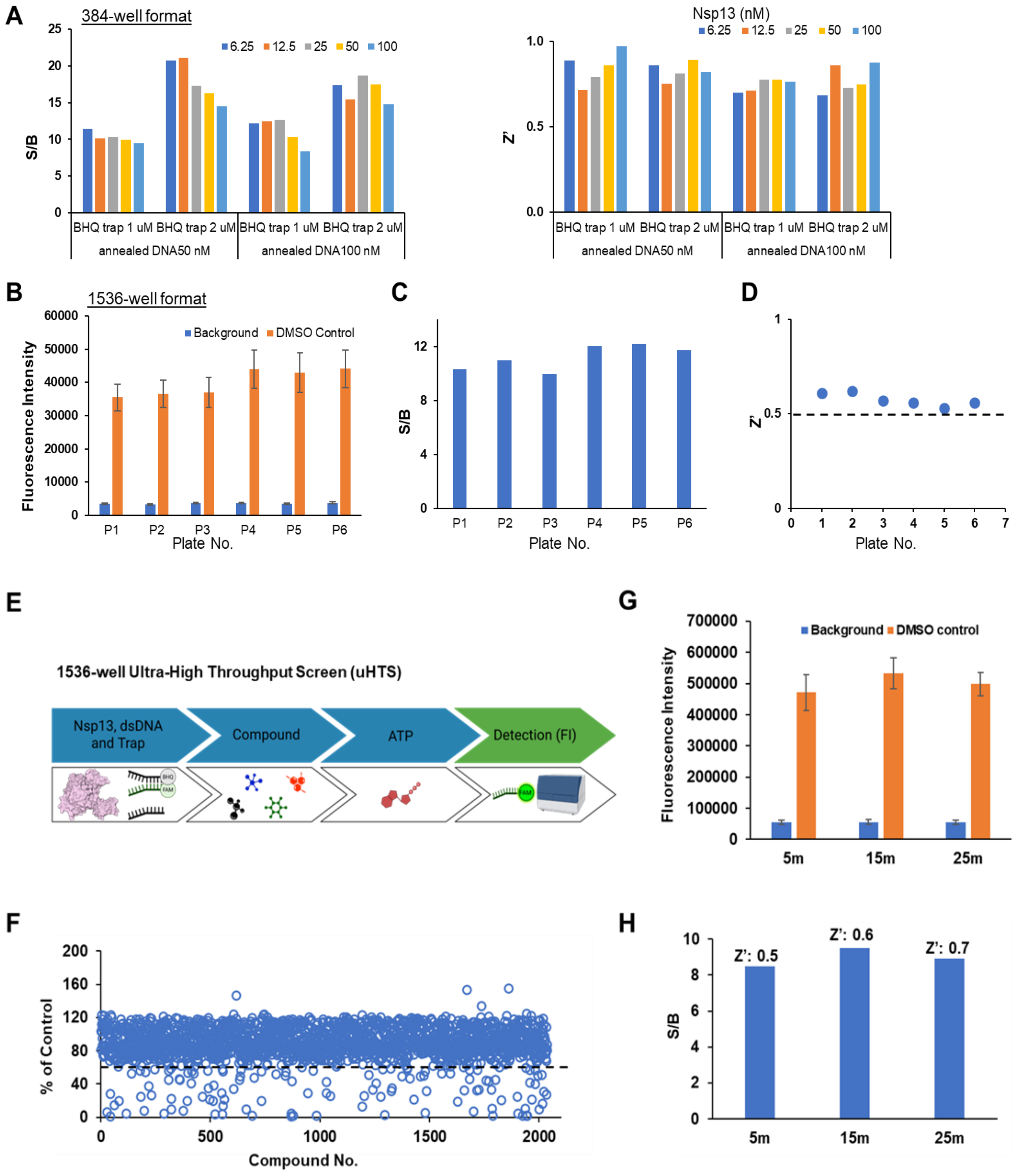

Supplemental Figure S1. FRET-based assay optimization and pilot screening of FDA-approved and bioactive compound library in 1536-well uHTS format.

(A) The fluorescence resonance energy transfer (FRET) assay that monitors nsp13 unwinding of FAM/BHQ dsDNA observed in 384-well plates. To determine the assay performance for HTS, the signal-to-background (S/B) ratio and the Z′ factor were calculated based on the following equations: S/B = μb/μf, where μb and μf are the FRET signals for unwound (b) FAM-ssDNA and annealed (f) FAM/BHQ-dsDNA substrate, respectively. The Z′ factor was calculated as: Z′ = 1 – (3 × SDb + 3 × SDf)/(μb-μf), where SDb and SDf are the standard deviations for unwound (b) and annealed (f) conditions. The Z′ factor reflects the quality of the assay and quantifies the suitability of a particular assay for use in HTS. A Z′ factor between 0.5 and 1.0 indicates an excellent assay for HTS. Different concentrations of nsp13 were tested in 384-well plate with S/B above 5 and Z' factors higher than 0.5, indicating the assay is robust in 384-well HTS format.

(B-D) Miniaturization of the assay into 1536-well uHTS format. The assay with optimized conditions in 384-well format was carried out in 1536-well plates. Fluorescence intensity signal from 6 1536-well plates was significantly higher in the Nsp13 reaction with ATP compared to that without ATP (background FI signal). All 6 1536-well plates showed S/B above 8 (C) and a Z' factor above 0.5 (D), indicating robustness of this assay in 1536-well uHTS format.

(E) Schematic representation of 1536-well uHTS screening. Figure created in BioRender. Goins, S. (2026) https://BioRender.com/3gff433

(F) The percentage of control is calculated as % of control = (FI compound-FI background)/(FI DMSO control-FI background) * 100 and plotted against compound ID. The potential positives are defined by the compound with % Control < 60 at 20 μM. From these, 74 compounds were selected for dose-response (DR) confirmatory screening.

(G) Fluorescent intensity signal at different time points in 1536-well format is stable, which is consistent with assay optimization data.

(H) The assay performance of DR plate is robust and consistent with primary uHTS with S/B > 8 and Z' > 0.5 at the tested time points.

Compound	IC_{50} [μM] ± SE	Reference
Doxorubicin (Adriamycin)	1.4 ± 0.1	[1],[2]
Mitoxantrone	2.3 ± 0.1	[2],[3]
Mitoxantrone HCl	2.5 ± 0.1	[2],[3]
Epirubicin HCl	3.1 ± 0.1	[4]
Daunorubicin HCl	3.4 ± 0.2	[5],[6]
Tariquidar	3.6 ± 0.1	[7]
ABT-263 (Navitoclax)	3.7 ± 0.6	[8],[9]
Candesartan Cilexetil	3.9 ± 0.5	[10],[11]
SGI-1027	4.1 ± 0.2	[12]
Venetoclax (ABT-199)	4.1 ± 0.2	[13],[14]
Voxtalisib (SAR245409)	4.2 ± 0.3	[15],[16]
Pranlukast	4.3 ± 0.5	[17],[18]
Avasimibe	5.6 ± 0.3	[19],[20]
CX-6258 HCl	5.6 ± 0.5	[21]
GW3965 HCl	5.6 ± 0.6	[22],[23]
Verteporfin	5.8 ± 0.5	[24],[25]
JTC-801	5.9 ± 0.9	[26]
Ursolic Acid	6.0 ± 0.4	[27],[28]
Montelukast Sodium	6.4 ± 0.2	[29]
Thonzonium Bromide	6.5 ± 0.5	[30]
IOWH032	6.8 ± 0.3	[31]
ZCL278	6.8 ± 0.6	[32],[33]
Alexidine HCl	6.8 ± 1.1	[34],[35]
Zotarolimus (ABT-578)	6.9 ± 0.5	[36],[37]
Deltarasin	7.0 ± 0.4	[38]
Closantel Sodium	7.1 ± 0.6	[39],[40]
ABT-737	7.1 ± 1.8	[41]
BMS-833923	7.3 ± 0.5	[42]
Crystal Violet	7.5 ± 0.4	[43]
Zinc Pyrithione	8.1 ± 0.6	[44]
Sabutoclax (BI-97C1)	8.2 ± 0.8	[45],[46]
TW-37	8.3 ± 0.7	[47]
SNS-314 Mesylate	9.3 ± 0.8	[48],[49]
4E1Rcat	9.3 ± 0.8	[50],[51]
TCID*	9.6 ± 0.8**	[52],[53]
BGT226 (NVP-BGT226)	9.6 ± 1.1	[54],[55]
PHT-427	10.2 ± 1.2	[56]
Nintedanib (BIBF 1120)	10.3 ± 0.5	[57],[58]
Ceritinib (LDK378)	10.5 ± 1.2	[59],[60]
Vitamin D2	11.4 ± 0.8	[61]

Tubacin	11.5 ± 1.0	[62],[63]
CAY10505	11.9 ± 2.1	[64],[65]
Sorafenib Tosylate	12.4 ± 2.6	[66],[67]
MRS 2578	12.5 ± 1.1	[68],[69]
Cisplatin	12.7 ± 2.7	[70]
GW4064	12.9 ± 0.6	[71]
Adapalene	13.3 ± 0.6	[72],[73]
Colistimethate Sodium	13.7 ± 1.2	[74]
OSU-03012 (AR-12)	14.1 ± 1.9	[75],[76]
XL147 analogue	14.3 ± 3.1	[77]
NSC 23766	14.8 ± 2.8	[78],[79]
SC75741	14.9 ± 2.0	[80],[81]
PR-619	15.2 ± 1.2	[82]
Otilonium Bromide	15.3 ± 1.1	[83],[84]
LDN-212854	16.1 ± 0.9	[85]
Evacetrapib (LY2484595)	16.1 ± 1.4	[86],[87]
VE-822	16.6 ± 1.2	[88],[89]
AT101	18.4 ± 2.5	[90],[91]
DCC-2036 (Rebastinib)	18.5 ± 2.5	[92],[93]
AMG-900	19.4 ± 2.1	[94],[95]
Azelnidipine	> 20	[96]
Linifanib (ABT-869)	> 20	[97],[98]
SRT1720	> 20	[99],[100]
Obatoclax Mesylate (GX15-070)	> 20	[101],[102]
BMS-794833	> 20	[103]
Tanshinone I	> 20	[104],[105]
CUDC-907	> 20	[106],[107]
Diminazene Aceturate	> 20	[108]
AZD3463	> 20	[109],[110]
Ehop-016	> 20	[111],[112]
WH-4-023	> 20	[113]
NH125	> 20	[114],[115]
Darapladib (SB-480848)	> 20	[116],[117]

Supplemental Table S1. Determination of IC_{50} values for selected compounds identified by uHTS. Dose-response enzymatic assays were used to calculate IC_{50} values for selected compounds. IC_{50} values are the average of three independent time points performed in quadruplicate. SE: Standard error. Details about the cellular targets of these compounds can be found in the associated references.* TCID: 4,5,6,7-Tetrachloroindan-1,3-dione. ** Included two more data sets to reduce uncertainty.

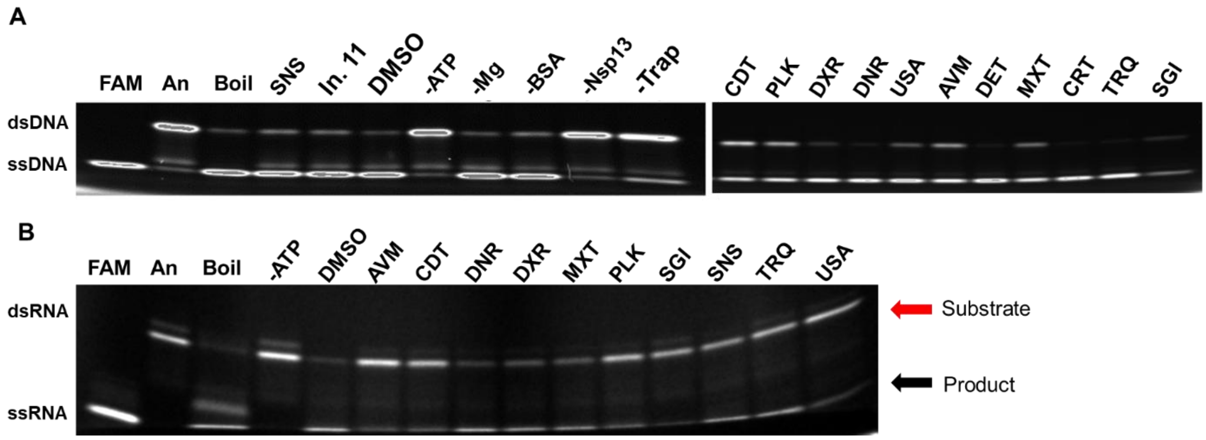

Supplemental Figure S2. Native-PAGE helicase assay to validate FRET-based nsp13 hits.
(A and B) Ten selected compounds exhibiting inhibition of nsp13 activity measured by FRET were tested in an orthogonal assay to test for inhibition directly. Nsp13 activity was tested in the presence of inhibitors at 20 µM on dsDNA (A) or dsRNA (B) substrates. Each nucleic acid duplex consisted of a 6-FAM labeled strand and an unlabeled complementary strand. Reactions were resolved by gel electrophoresis under native conditions.

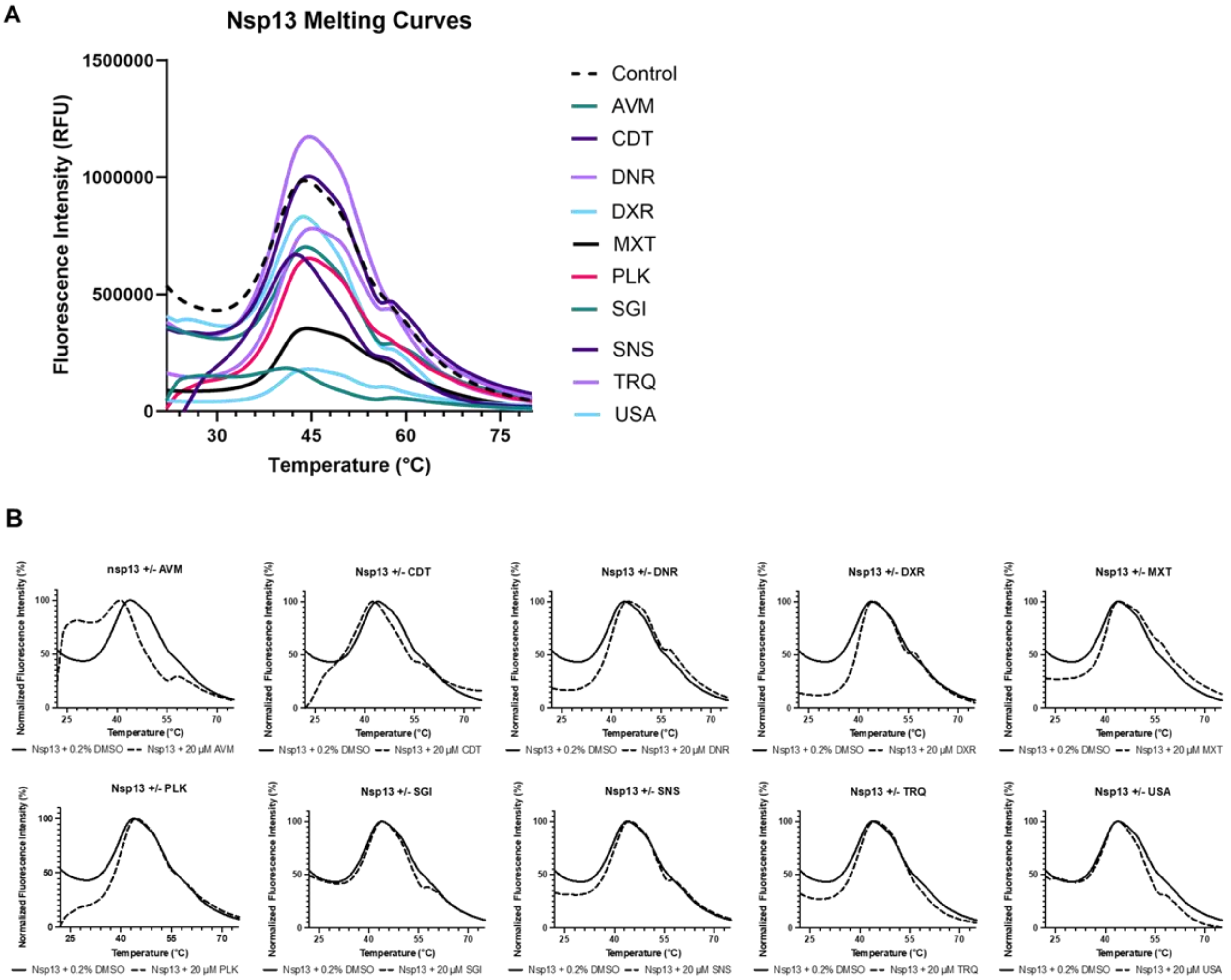

Supplemental Figure S3. Raw melting profiles of nsp13 with inhibitors assessed by DSF.
(A) Nsp13 in the presence of DMSO (control) is shown as a black dotted line. Nsp13 in the presence of 20 μM inhibitor is shown as a solid, colored line. Each sample was tested in quadruplicates, and the plot for each sample represents the mean for each data point.
(B) Each curve represents the mean of 4 independent experiments. These data have been baseline subtracted (inhibitor + dye, no nsp13) and normalized by fluorescence intensity. Melting temperatures were determined by the temperature corresponding to the maximum of the first derivative of these melting profiles.

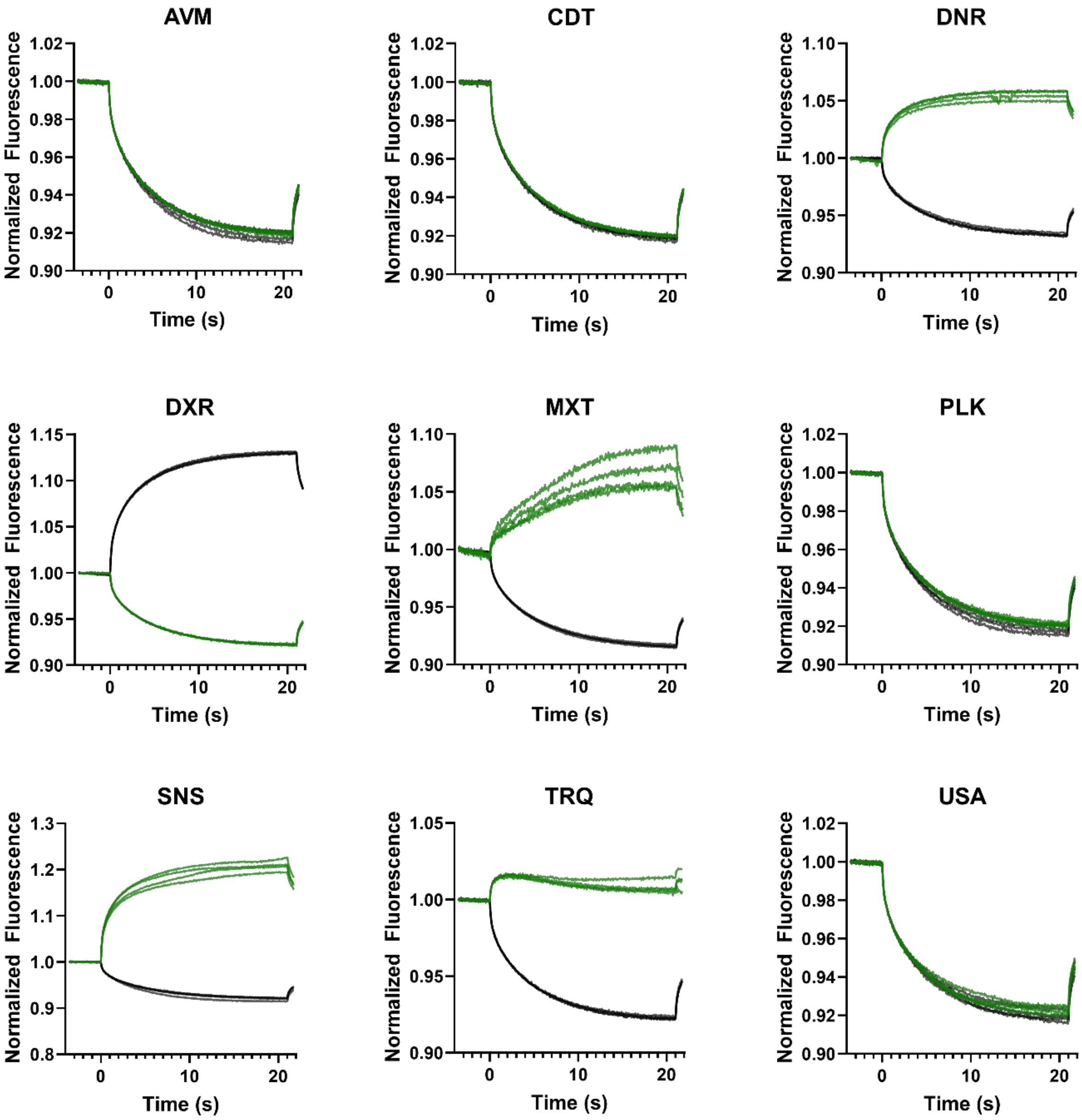

Supplemental Figure S4. Thermophoretic traces of FAM-labeled dsRNA in the absence and presence of nsp13 inhibitors. dsRNA thermophoresis is shown in the absence (black), or presence (green) of nsp13 inhibitors. Qualitative differences in the thermophoretic traces in the presence of inhibitors correspond to differences in the diffusion of the FAM-labeled dsRNA. This is inferred to be a consequence of dsRNA/inhibitor binding. DNR, DXR, MXT, SNS, and TRQ interact with the nucleic acid duplex (shift in the thermophoretic traces). SGI was not reported due to non-specific changes in the FAM-labeled dsRNA fluorescence intensity in the presence of the inhibitor. Each condition was tested in quadruplicates.

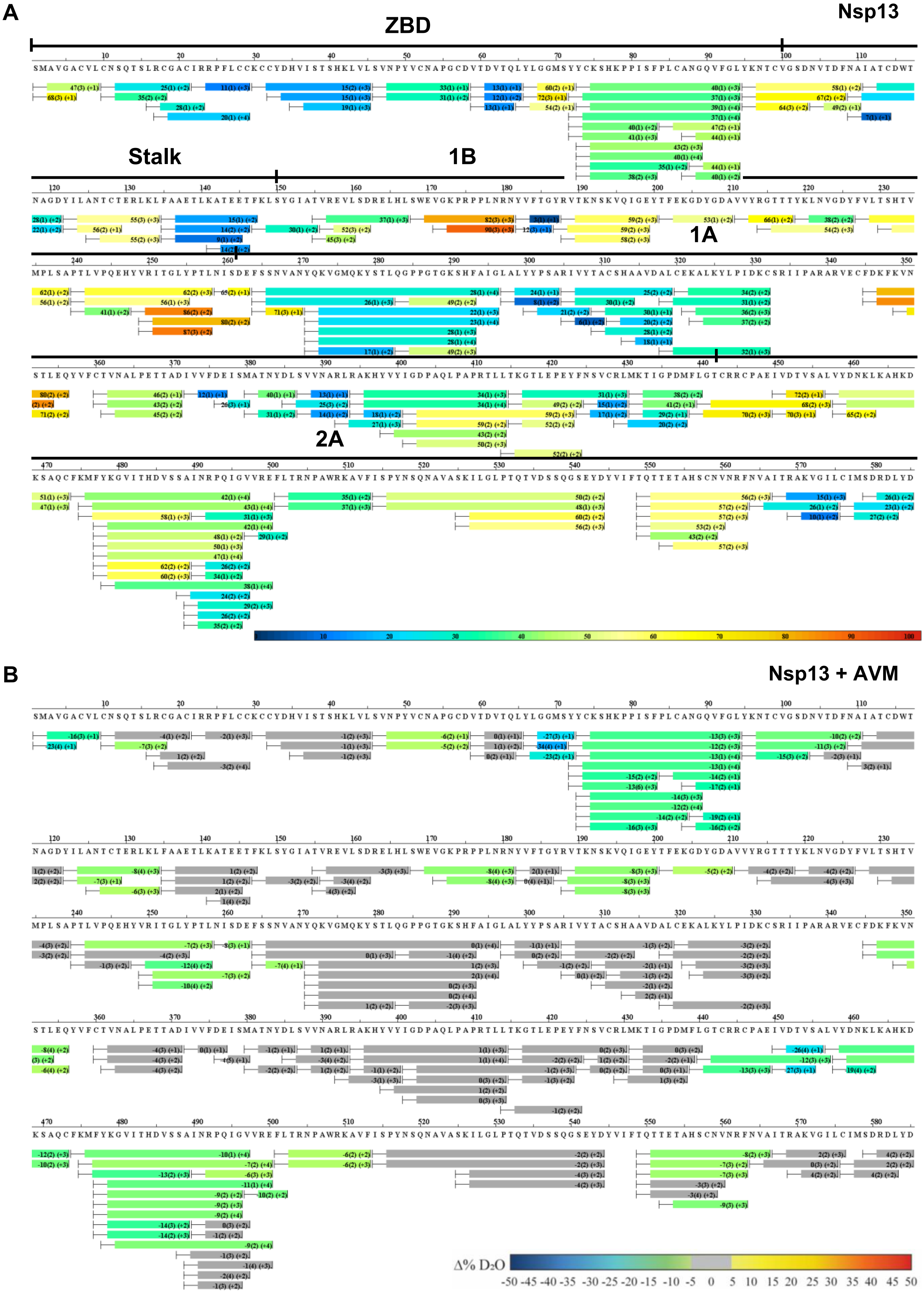
A
Nsp13
ZBD
Stalk
1B
1A
2A
B
Nsp13 + AVM
Δ% D2O

C **Nsp13 + CDT**

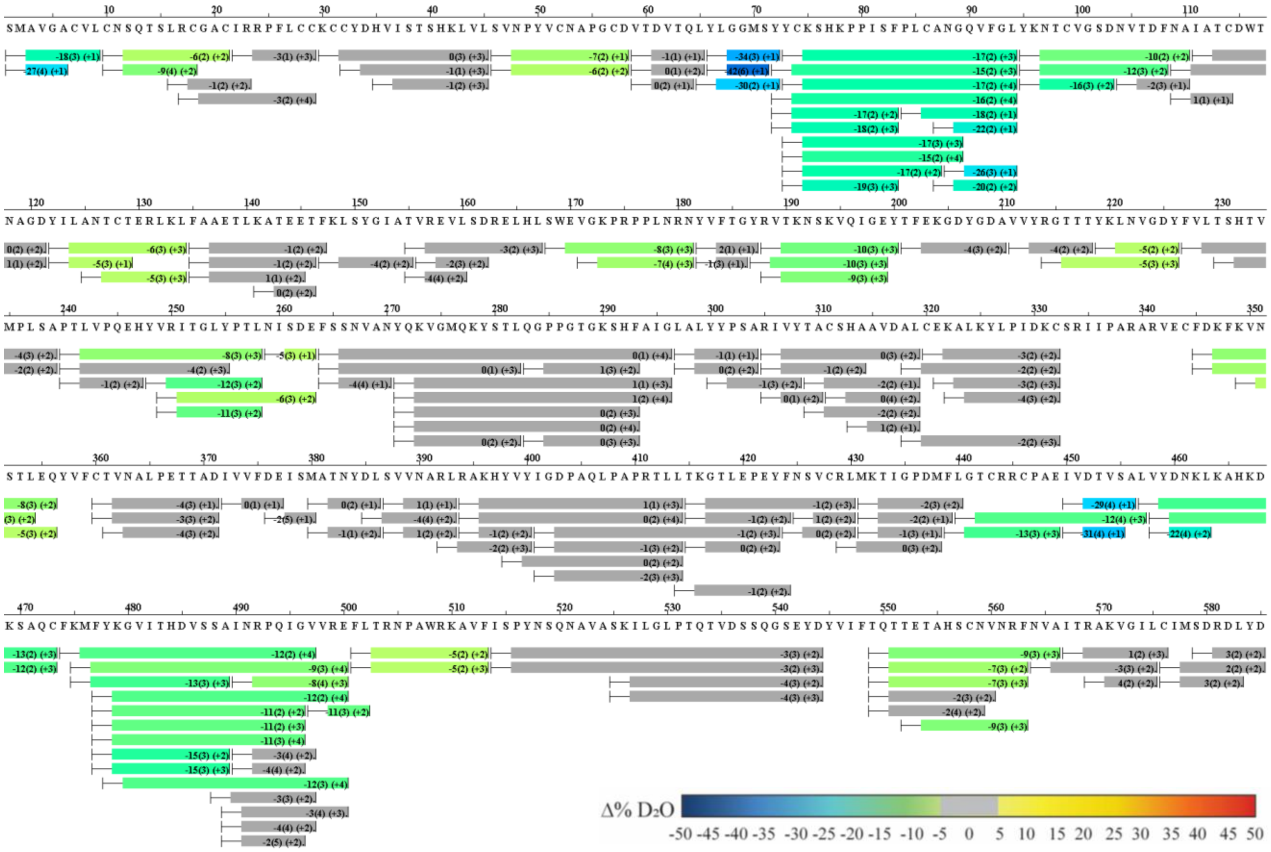

D **Nsp13 + PLK**

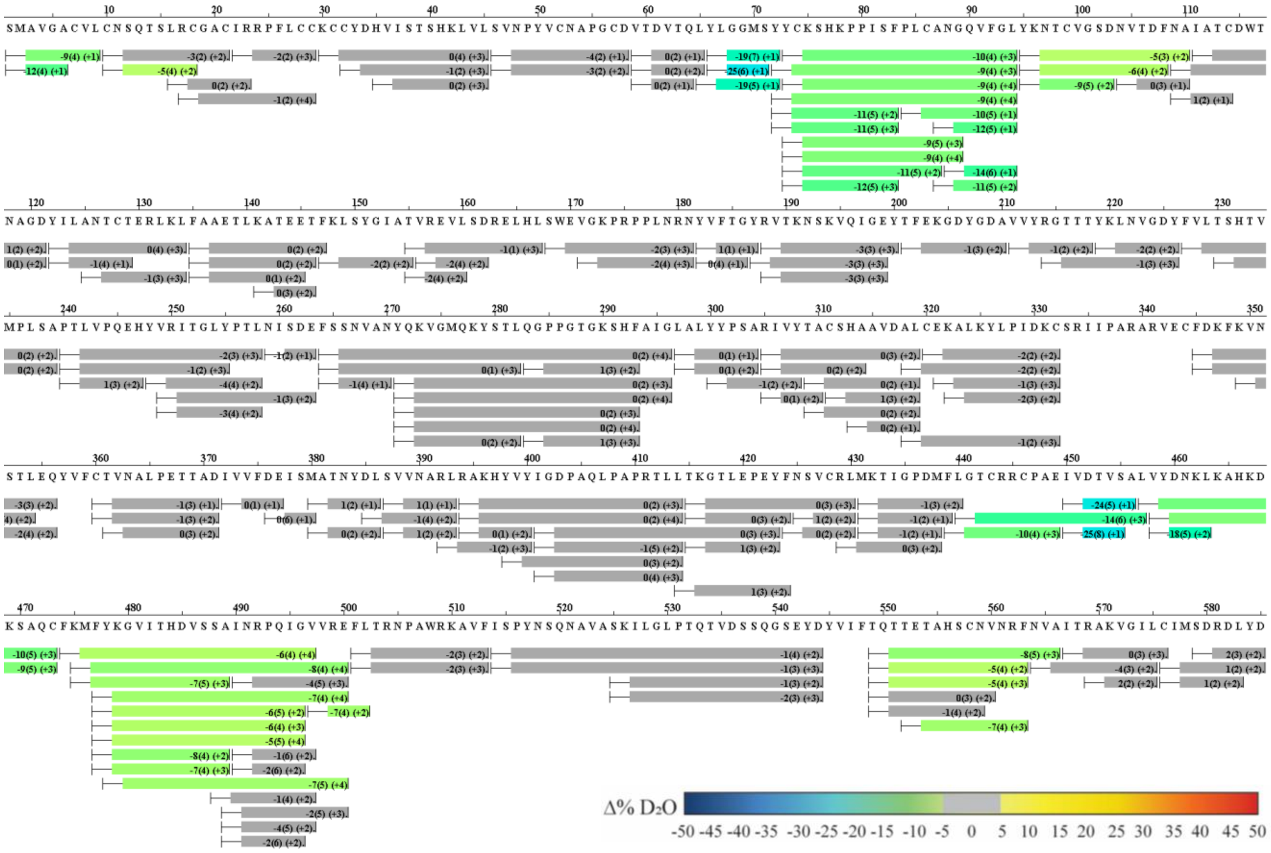

E

Nsp13 + USA

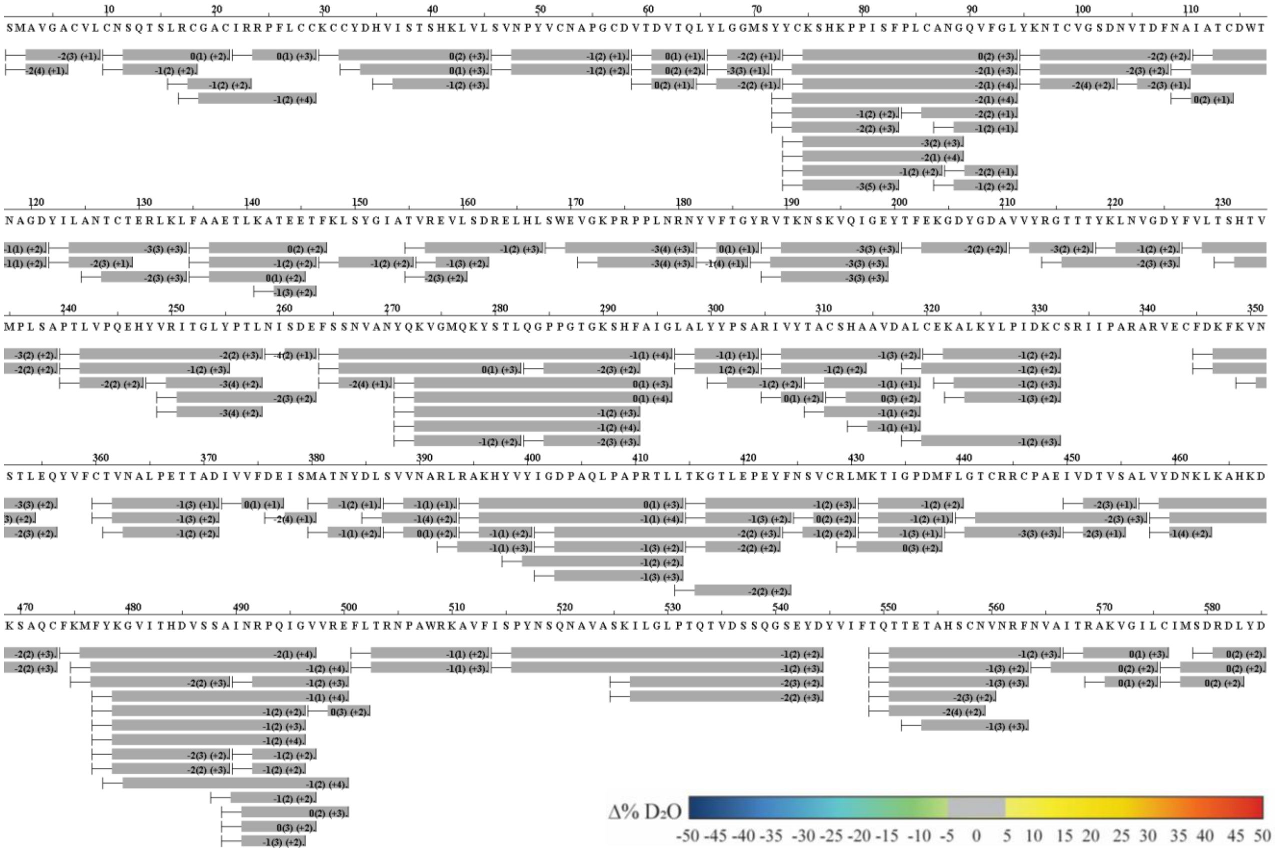

Supplemental Figure S5. Nsp13 deuterium exchange perturbation plots in the presence and absence of compounds.

(A) Unliganded nsp13. Values shown for each peptide indicate the %D, standard deviation, and peptide charge state, respectively. Colorimetric scale represents 0-100% deuterium incorporation. ZBD: Zinc Binding Domain, 1B: beta-barrel 1B domain, 1A: RecA-like domain 1A, 2A: RecA-like domain 2A.

(B-E) Colorimetric scale (-50% to 50%) represents the difference in the rate of deuterium exchange between ligand-treated and apo protein. Peptides with negative values indicate regions where ligand-treated samples showed protection from exchange relative to the apo protein.

REFERENCES

[1] Tewey KM, Rowe TC, Yang L, Halligan BD, Liu LF. Adriamycin-induced DNA damage mediated by mammalian DNA topoisomerase II. Science. 1984;226:466-8.
[2] Wu CC, Li YC, Wang YR, Li TK, Chan NL. On the structural basis and design guidelines for type II topoisomerase-targeting anticancer drugs. Nucleic Acids Res. 2013;41:10630-40.
[3] Hasinoff BB, Liang H, Wu X, Guziec LJ, Guziec FS, Jr., Marshall K, et al. The structure-based design, synthesis and biological evaluation of DNA-binding bisintercalating bisanthrapyrazole anticancer compounds. Bioorg Med Chem. 2008;16:3959-68.
[4] Plosker GL, Faulds D. Epirubicin. A review of its pharmacodynamic and pharmacokinetic properties, and therapeutic use in cancer chemotherapy. Drugs. 1993;45:788-856.
[5] Lehmann M, Vilar Kde S, Franco A, Reguly ML, Rodrigues de Andrade HH. Activity of topoisomerase inhibitors daunorubicin, idarubicin, and aclarubicin in the Drosophila Somatic Mutation and Recombination Test. Environ Mol Mutagen. 2004;43:250-7.
[6] Arcamone F, Animati F, Bigioni M, Capranico G, Caserini C, Cipollone A, et al. Configurational requirements of the sugar moiety for the pharmacological activity of anthracycline disaccharides. Biochem Pharmacol. 1999;57:1133-9.
[7] Choo EF, Kurnik D, Muszkat M, Ohkubo T, Shay SD, Higginbotham JN, et al. Differential in vivo sensitivity to inhibition of P-glycoprotein located in lymphocytes, testes, and the blood-brain barrier. J Pharmacol Exp Ther. 2006;317:1012-8.
[8] Tse C, Shoemaker AR, Adickes J, Anderson MG, Chen J, Jin S, et al. ABT-263: a potent and orally bioavailable Bcl-2 family inhibitor. Cancer Res. 2008;68:3421-8.
[9] Bulanova D, Ianevski A, Bugai A, Akimov Y, Kuivanen S, Paavilainen H, et al. Antiviral Properties of Chemical Inhibitors of Cellular Anti-Apoptotic Bcl-2 Proteins. Viruses. 2017;9.
[10] McClellan KJ, Goa KL. Candesartan cilexetil. A review of its use in essential hypertension. Drugs. 1998;56:847-69.
[11] Gavras H. Update on the clinical pharmacology of candesartan cilexetil. Am J Hypertens. 2000;13:25S-30S.
[12] Sun N, Zhang J, Zhang C, Zhao B, Jiao A. DNMTs inhibitor SGI-1027 induces apoptosis in Huh7 human hepatocellular carcinoma cells. Oncol Lett. 2018;16:5799-806.
[13] Davids MS, Letai A. ABT-199: taking dead aim at BCL-2. Cancer Cell. 2013;23:139-41.
[14] Tamaki H, Harashima N, Hiraki M, Arichi N, Nishimura N, Shiina H, et al. Bcl-2 family inhibition sensitizes human prostate cancer cells to docetaxel and promotes unexpected apoptosis under caspase-9 inhibition. Oncotarget. 2014;5:11399-412.
[15] Prasad G, Sottero T, Yang X, Mueller S, James CD, Weiss WA, et al. Inhibition of PI3K/mTOR pathways in glioblastoma and implications for combination therapy with temozolomide. Neuro Oncol. 2011;13:384-92.
[16] Yu P, Laird AD, Du X, Wu J, Won KA, Yamaguchi K, et al. Characterization of the activity of the PI3K/mTOR inhibitor XL765 (SAR245409) in tumor models with diverse genetic alterations affecting the PI3K pathway. Mol Cancer Ther. 2014;13:1078-91.
[17] Liu YC, Khawaja AM, Rogers DF. Effects of the cysteinyl leukotriene receptor antagonists pranlukast and zafirlukast on tracheal mucus secretion in ovalbumin-sensitized guinea-pigs in vitro. Br J Pharmacol. 1998;124:563-71.
[18] Yu S, Chen X, Li X, Yan J, Jiang Y. Neuroprotective effects of CysLTR antagonist on Streptococcus pneumoniae-induced meningitis in rats. Exp Ther Med. 2022;24:443.

[19] Burnett JR, Wilcox LJ, Telford DE, Kleinstiver SJ, Barrett PHR, Newton RS, et al. Inhibition of ACAT by avasimibe decreases both VLDL and LDL apolipoprotein B production in miniature pigs. Journal of Lipid Research. 1999;40:1317-27.
[20] Llaverías G, Laguna JC, Alegret M. Pharmacology of the AC AT Inhibitor Avasimibe (CI-1011). Cardiovascular Drug Reviews. 2006;21:33-50.
[21] Haddach M, Michaux J, Schwaebe MK, Pierre F, O'Brien SE, Borsan C, et al. Discovery of CX-6258. A Potent, Selective, and Orally Efficacious pan-Pim Kinases Inhibitor. ACS Med Chem Lett. 2012;3:135-9.
[22] Collins JL, Fivush AM, Watson MA, Galardi CM, Lewis MC, Moore LB, et al. Identification of a nonsteroidal liver X receptor agonist through parallel array synthesis of tertiary amines. J Med Chem. 2002;45:1963-6.
[23] Leik CE, Carson NL, Hennan JK, Basso MD, Liu QY, Crandall DL, et al. GW3965, a synthetic liver X receptor (LXR) agonist, reduces angiotensin II-mediated pressor responses in Sprague-Dawley rats. Br J Pharmacol. 2007;151:450-6.
[24] Schmidt-Erfurth U, Hasan T. Mechanisms of action of photodynamic therapy with verteporfin for the treatment of age-related macular degeneration. Surv Ophthalmol. 2000;45:195-214.
[25] Morishita T, Hayakawa F, Sugimoto K, Iwase M, Yamamoto H, Hirano D, et al. The photosensitizer verteporfin has light-independent anti-leukemic activity for Ph-positive acute lymphoblastic leukemia and synergistically works with dasatinib. Oncotarget. 2016;7:56241-52.
[26] Yamada H, Nakamoto H, Suzuki Y, Ito T, Aisaka K. Pharmacological profiles of a novel opioid receptor-like1 (ORL(1)) receptor antagonist, JTC-801. Br J Pharmacol. 2002;135:323-32.
[27] He X, Liu RH. Triterpenoids isolated from apple peels have potent antiproliferative activity and may be partially responsible for apple's anticancer activity. J Agric Food Chem. 2007;55:4366-70.
[28] Wozniak L, Skapska S, Marszalek K. Ursolic Acid--A Pentacyclic Triterpenoid with a Wide Spectrum of Pharmacological Activities. Molecules. 2015;20:20614-41.
[29] Heise CE, O'Dowd BF, Figueroa DJ, Sawyer N, Nguyen T, Im DS, et al. Characterization of the human cysteinyl leukotriene 2 receptor. J Biol Chem. 2000;275:30531-6.
[30] Chan CY, Prudom C, Raines SM, Charkhzarrin S, Melman SD, De Haro LP, et al. Inhibitors of V-ATPase proton transport reveal uncoupling functions of tether linking cytosolic and membrane domains of V0 subunit a (Vph1p). J Biol Chem. 2012;287:10236-50.
[31] Cui G, Khazanov N, Stauffer BB, Infield DT, Imhoff BR, Senderowitz H, et al. Potentiators exert distinct effects on human, murine, and Xenopus CFTR. Am J Physiol Lung Cell Mol Physiol. 2016;311:L192-207.
[32] Chou YY, Cuevas C, Carocci M, Stubbs SH, Ma M, Cureton DK, et al. Identification and Characterization of a Novel Broad-Spectrum Virus Entry Inhibitor. J Virol. 2016;90:4494-510.
[33] Friesland A, Zhao Y, Chen YH, Wang L, Zhou H, Lu Q. Small molecule targeting Cdc42-intersectin interaction disrupts Golgi organization and suppresses cell motility. Proc Natl Acad Sci U S A. 2013;110:1261-6.
[34] Zorko M, Jerala R. Alexidine and chlorhexidine bind to lipopolysaccharide and lipoteichoic acid and prevent cell activation by antibiotics. J Antimicrob Chemother. 2008;62:730-7.
[35] Yip KW, Ito E, Mao X, Au PY, Hedley DW, Mocanu JD, et al. Potential use of alexidine dihydrochloride as an apoptosis-promoting anticancer agent. Mol Cancer Ther. 2006;5:2234-40.
[36] Collingwood R, Gibson L, Sedlik S, Virmani R, Carter AJ. Stent-based delivery of ABT-578 via a phosphorylcholine surface coating reduces neointimal formation in the porcine coronary model. Catheter Cardiovasc Interv. 2005;65:227-32.

[37] Chen YW, Smith ML, Sheets M, Ballaron S, Trevillyan JM, Burke SE, et al. Zotarolimus, a novel sirolimus analogue with potent anti-proliferative activity on coronary smooth muscle cells and reduced potential for systemic immunosuppression. J Cardiovasc Pharmacol. 2007;49:228-35.
[38] Zimmermann G, Papke B, Ismail S, Vartak N, Chandra A, Hoffmann M, et al. Small molecule inhibition of the KRAS-PDEdelta interaction impairs oncogenic KRAS signalling. Nature. 2013;497:638-42.
[39] AlAmri MA, Kadri H, Alderwick LJ, Simpkins NS, Mehellou Y. Rafoxanide and Closantel Inhibit SPAK and OSR1 Kinases by Binding to a Highly Conserved Allosteric Site on Their C-terminal Domains. ChemMedChem. 2017;12:639-45.
[40] Sie ZL, Li RY, Sampurna BP, Hsu PJ, Liu SC, Wang HD, et al. WNK1 Kinase Stimulates Angiogenesis to Promote Tumor Growth and Metastasis. Cancers (Basel). 2020;12.
[41] Zhou H, Chen J, Meagher JL, Yang CY, Aguilar A, Liu L, et al. Design of Bcl-2 and Bcl-xL inhibitors with subnanomolar binding affinities based upon a new scaffold. J Med Chem. 2012;55:4664-82.
[42] AlMuraikhi N, Almasoud N, Binhamdan S, Younis G, Ali D, Manikandan M, et al. Hedgehog Signaling Inhibition by Smoothened Antagonist BMS-833923 Reduces Osteoblast Differentiation and Ectopic Bone Formation of Human Skeletal (Mesenchymal) Stem Cells. Stem Cells Int. 2019;2019:3435901.
[43] Maley AM, Arbiser JL. Gentian violet: a 19th century drug re-emerges in the 21st century. Exp Dermatol. 2013;22:775-80.
[44] Srivastava G, Matta A, Fu G, Somasundaram RT, Datti A, Walfish PG, et al. Anticancer activity of pyrithione zinc in oral cancer cells identified in small molecule screens and xenograft model: Implications for oral cancer therapy. Mol Oncol. 2015;9:1720-35.
[45] Wei J, Stebbins JL, Kitada S, Dash R, Placzek W, Rega MF, et al. BI-97C1, an optically pure Apogossypol derivative as pan-active inhibitor of antiapoptotic B-cell lymphoma/leukemia-2 (Bcl-2) family proteins. J Med Chem. 2010;53:4166-76.
[46] Hu Y, Yagüe E, Zhao J, Wang L, Bai J, Yang Q, et al. Sabutoclax, pan-active BCL-2 protein family antagonist, overcomes drug resistance and eliminates cancer stem cells in breast cancer. Cancer Letters. 2018;423:47-59.
[47] Zeitlin BD, Joo E, Dong Z, Warner K, Wang G, Nikolovska-Coleska Z, et al. Antiangiogenic effect of TW37, a small-molecule inhibitor of Bcl-2. Cancer Res. 2006;66:8698-706.
[48] Oslob JD, Romanowski MJ, Allen DA, Baskaran S, Bui M, Elling RA, et al. Discovery of a potent and selective aurora kinase inhibitor. Bioorg Med Chem Lett. 2008;18:4880-4.
[49] Arbitrario JP, Belmont BJ, Evanchik MJ, Flanagan WM, Fucini RV, Hansen SK, et al. SNS-314, a pan-Aurora kinase inhibitor, shows potent anti-tumor activity and dosing flexibility in vivo. Cancer Chemother Pharmacol. 2010;65:707-17.
[50] Cencic R, Hall DR, Robert F, Du Y, Min J, Li L, et al. Reversing chemoresistance by small molecule inhibition of the translation initiation complex eIF4F. Proc Natl Acad Sci U S A. 2011;108:1046-51.
[51] Fang C, Xie H, Zhao J, Wang W, Hou H, Zhang B, et al. eIF4E-eIF4G complex inhibition synergistically enhances the effect of sorafenib in hepatocellular carcinoma. Anticancer Drugs. 2021;32:822-8.
[52] Liu Y, Lashuel HA, Choi S, Xing X, Case A, Ni J, et al. Discovery of inhibitors that elucidate the role of UCH-L1 activity in the H1299 lung cancer cell line. Chem Biol. 2003;10:837-46.

[53] de Juan-Sanz J, Nunez E, Lopez-Corcuera B, Aragon C. Constitutive endocytosis and turnover of the neuronal glycine transporter GlyT2 is dependent on ubiquitination of a C-terminal lysine cluster. PLoS One. 2013;8:e58863.
[54] Chang KY, Tsai SY, Wu CM, Yen CJ, Chuang BF, Chang JY. Novel phosphoinositide 3-kinase/mTOR dual inhibitor, NVP-BGT226, displays potent growth-inhibitory activity against human head and neck cancer cells in vitro and in vivo. Clin Cancer Res. 2011;17:7116-26.
[55] Markman B, Tabernero J, Krop I, Shapiro GI, Siu L, Chen LC, et al. Phase I safety, pharmacokinetic, and pharmacodynamic study of the oral phosphatidylinositol-3-kinase and mTOR inhibitor BGT226 in patients with advanced solid tumors. Ann Oncol. 2012;23:2399-408.
[56] Meuillet EJ, Zuohe S, Lemos R, Ihle N, Kingston J, Watkins R, et al. Molecular pharmacology and antitumor activity of PHT-427, a novel Akt/phosphatidylinositide-dependent protein kinase 1 pleckstrin homology domain inhibitor. Mol Cancer Ther. 2010;9:706-17.
[57] Hilberg F, Roth GJ, Krssak M, Kautschitsch S, Sommergruber W, Tontsch-Grunt U, et al. BIBF 1120: triple angiokinase inhibitor with sustained receptor blockade and good antitumor efficacy. Cancer Res. 2008;68:4774-82.
[58] Herman D, Ghazipura M, Barnes H, Macrea M, Knight SL, Silver RM, et al. Nintedanib Therapy Alone and Combined with Mycophenolate in Patients with Systemic Sclerosis-associated Interstitial Lung Disease: Systematic Reviews and Meta-analysis. Ann Am Thorac Soc. 2024;21:474-85.
[59] Marsilje TH, Pei W, Chen B, Lu W, Uno T, Jin Y, et al. Synthesis, structure-activity relationships, and in vivo efficacy of the novel potent and selective anaplastic lymphoma kinase (ALK) inhibitor 5-chloro-N2-(2-isopropoxy-5-methyl-4-(piperidin-4-yl)phenyl)-N4-(2-(isopropylsulfonyl)phenyl)pyrimidine-2,4-diamine (LDK378) currently in phase 1 and phase 2 clinical trials. J Med Chem. 2013;56:5675-90.
[60] Fontana D, Ceccon M, Gambacorti-Passerini C, Mologni L. Activity of second-generation ALK inhibitors against crizotinib-resistant mutants in an NPM-ALK model compared to EML4-ALK. Cancer Med. 2015;4:953-65.
[61] Houghton LA, Vieth R. The case against ergocalciferol (vitamin D2) as a vitamin supplement. Am J Clin Nutr. 2006;84:694-7.
[62] Cole PA. Chemical probes for histone-modifying enzymes. Nat Chem Biol. 2008;4:590-7.
[63] Chen S, Owens GC, Makarenkova H, Edelman DB. HDAC6 regulates mitochondrial transport in hippocampal neurons. PLoS One. 2010;5:e10848.
[64] Pomel V, Klicic J, Covini D, Church DD, Shaw JP, Roulin K, et al. Furan-2-ylmethylene thiazolidinediones as novel, potent, and selective inhibitors of phosphoinositide 3-kinase gamma. J Med Chem. 2006;49:3857-71.
[65] Sun YY, Lin SH, Lin HC, Hung CC, Wang CY, Lin YC, et al. Cell type-specific dependency on the PI3K/Akt signaling pathway for the endogenous Epo and VEGF induction by baicalein in neurons versus astrocytes. PLoS One. 2013;8:e69019.
[66] Wilhelm SM, Carter C, Tang L, Wilkie D, McNabola A, Rong H, et al. BAY 43-9006 exhibits broad spectrum oral antitumor activity and targets the RAF/MEK/ERK pathway and receptor tyrosine kinases involved in tumor progression and angiogenesis. Cancer Res. 2004;64:7099-109.
[67] Dixon SJ, Patel DN, Welsch M, Skouta R, Lee ED, Hayano M, et al. Pharmacological inhibition of cystine-glutamate exchange induces endoplasmic reticulum stress and ferroptosis. Elife. 2014;3:e02523.

[68] Mamedova LK, Joshi BV, Gao ZG, von Kugelgen I, Jacobson KA. Diisothiocyanate derivatives as potent, insurmountable antagonists of P2Y6 nucleotide receptors. Biochem Pharmacol. 2004;67:1763-70.
[69] Sil P, Hayes CP, Reaves BJ, Breen P, Quinn S, Sokolove J, et al. P2Y6 Receptor Antagonist MRS2578 Inhibits Neutrophil Activation and Aggregated Neutrophil Extracellular Trap Formation Induced by Gout-Associated Monosodium Urate Crystals. J Immunol. 2017;198:428-42.
[70] Zhang L, Zheng Y, Callahan B, Belfort M, Liu Y. Cisplatin inhibits protein splicing, suggesting inteins as therapeutic targets in mycobacteria. J Biol Chem. 2011;286:1277-82.
[71] Watanabe M, Horai Y, Houten SM, Morimoto K, Sugizaki T, Arita E, et al. Lowering bile acid pool size with a synthetic farnesoid X receptor (FXR) agonist induces obesity and diabetes through reduced energy expenditure. J Biol Chem. 2011;286:26913-20.
[72] Ocker M, Herold C, Ganslmayer M, Hahn EG, Schuppan D. The synthetic retinoid adapalene inhibits proliferation and induces apoptosis in colorectal cancer cells in vitro. Int J Cancer. 2003;107:453-9.
[73] Tenaud I, Khammari A, Dreno B. In vitro modulation of TLR-2, CD1d and IL-10 by adapalene on normal human skin and acne inflammatory lesions. Exp Dermatol. 2007;16:500-6.
[74] Li J, Nation RL, Milne RW, Turnidge JD, Coulthard K. Evaluation of colistin as an agent against multi-resistant Gram-negative bacteria. Int J Antimicrob Agents. 2005;25:11-25.
[75] Zhu J, Huang JW, Tseng PH, Yang YT, Fowble J, Shiau CW, et al. From the cyclooxygenase-2 inhibitor celecoxib to a novel class of 3-phosphoinositide-dependent protein kinase-1 inhibitors. Cancer Res. 2004;64:4309-18.
[76] Johnson AJ, Smith LL, Zhu J, Heerema NA, Jefferson S, Mone A, et al. A novel celecoxib derivative, OSU03012, induces cytotoxicity in primary CLL cells and transformed B-cell lymphoma cell line via a caspase- and Bcl-2-independent mechanism. Blood. 2005;105:2504-9.
[77] Foster P, Yamaguchi K, Hsu PP, Qian F, Du X, Wu J, et al. The Selective PI3K Inhibitor XL147 (SAR245408) Inhibits Tumor Growth and Survival and Potentiates the Activity of Chemotherapeutic Agents in Preclinical Tumor Models. Mol Cancer Ther. 2015;14:931-40.
[78] Rao JN, Liu SV, Zou T, Liu L, Xiao L, Zhang X, et al. Rac1 promotes intestinal epithelial restitution by increasing Ca2+ influx through interaction with phospholipase C-(gamma)1 after wounding. Am J Physiol Cell Physiol. 2008;295:C1499-509.
[79] Vallon M, Rohde F, Janssen KP, Essler M. Tumor endothelial marker 5 expression in endothelial cells during capillary morphogenesis is induced by the small GTPase Rac and mediates contact inhibition of cell proliferation. Exp Cell Res. 2010;316:412-21.
[80] Leban J, Baierl M, Mies J, Trentinaglia V, Rath S, Kronthaler K, et al. A novel class of potent NF-kappaB signaling inhibitors. Bioorg Med Chem Lett. 2007;17:5858-62.
[81] Ehrhardt C, Ruckle A, Hrincius ER, Haasbach E, Anhlan D, Ahmann K, et al. The NF-kappaB inhibitor SC75741 efficiently blocks influenza virus propagation and confers a high barrier for development of viral resistance. Cell Microbiol. 2013;15:1198-211.
[82] Seiberlich V, Goldbaum O, Zhukareva V, Richter-Landsberg C. The small molecule inhibitor PR-619 of deubiquitinating enzymes affects the microtubule network and causes protein aggregate formation in neural cells: implications for neurodegenerative diseases. Biochim Biophys Acta. 2012;1823:2057-68.
[83] Martin MT, Hove-Madsen L, Jimenez M. Otilonium bromide inhibits muscle contractions via L-type calcium channels in the rat colon. Neurogastroenterol Motil. 2004;16:175-83.

[84] Strege PR, Sha L, Beyder A, Bernard CE, Perez-Reyes E, Evangelista S, et al. T-type Ca(2+) channel modulation by otilonium bromide. Am J Physiol Gastrointest Liver Physiol. 2010;298:G706-13.
[85] Mohedas AH, Xing X, Armstrong KA, Bullock AN, Cuny GD, Yu PB. Development of an ALK2-biased BMP type I receptor kinase inhibitor. ACS Chem Biol. 2013;8:1291-302.
[86] Cao G, Beyer TP, Zhang Y, Schmidt RJ, Chen YQ, Cockerham SL, et al. Evacetrapib is a novel, potent, and selective inhibitor of cholesteryl ester transfer protein that elevates HDL cholesterol without inducing aldosterone or increasing blood pressure. J Lipid Res. 2011;52:2169-76.
[87] Dong B, Singh AB, Fung C, Kan K, Liu J. CETP inhibitors downregulate hepatic LDL receptor and PCSK9 expression in vitro and in vivo through a SREBP2 dependent mechanism. Atherosclerosis. 2014;235:449-62.
[88] Charrier JD, Durrant SJ, Golec JM, Kay DP, Knegtel RM, MacCormick S, et al. Discovery of potent and selective inhibitors of ataxia telangiectasia mutated and Rad3 related (ATR) protein kinase as potential anticancer agents. J Med Chem. 2011;54:2320-30.
[89] Fokas E, Prevo R, Pollard JR, Reaper PM, Charlton PA, Cornelissen B, et al. Targeting ATR in vivo using the novel inhibitor VE-822 results in selective sensitization of pancreatic tumors to radiation. Cell Death Dis. 2012;3:e441.
[90] Kline MP, Rajkumar SV, Timm MM, Kimlinger TK, Haug JL, Lust JA, et al. R-(-)-gossypol (AT-101) activates programmed cell death in multiple myeloma cells. Exp Hematol. 2008;36:568-76.
[91] Fiveash JB, Ye X, Peerboom DM, Mikkelsen T, Chowdhary S, Rosenfeld M, et al. Clinical trials of R-(-)-gossypol (AT-101) in newly diagnosed and recurrent glioblastoma: NABTT 0602 and NABTT 0702. PLoS One. 2024;19:e0291128.
[92] Eide CA, Adrian LT, Tyner JW, Mac Partlin M, Anderson DJ, Wise SC, et al. The ABL switch control inhibitor DCC-2036 is active against the chronic myeloid leukemia mutant BCR-ABLT315I and exhibits a narrow resistance profile. Cancer Res. 2011;71:3189-95.
[93] Shen Y, Shi X, Pan J. The conformational control inhibitor of tyrosine kinases DCC-2036 is effective for imatinib-resistant cells expressing T674I FIP1L1-PDGFRalpha. PLoS One. 2013;8:e73059.
[94] Payton M, Bush TL, Chung G, Ziegler B, Eden P, McElroy P, et al. Preclinical evaluation of AMG 900, a novel potent and highly selective pan-aurora kinase inhibitor with activity in taxane-resistant tumor cell lines. Cancer Res. 2010;70:9846-54.
[95] Fischer N, Seo EJ, Klinger A, Fleischer E, Efferth T. AMG900 as novel inhibitor of the translationally controlled tumor protein. Chem Biol Interact. 2021;334:109349.
[96] Chen BL, Zhang YZ, Luo JQ, Zhang W. Clinical use of azelnidipine in the treatment of hypertension in Chinese patients. Ther Clin Risk Manag. 2015;11:309-18.
[97] Albert DH, Tapang P, Magoc TJ, Pease LJ, Reuter DR, Wei RQ, et al. Preclinical activity of ABT-869, a multitargeted receptor tyrosine kinase inhibitor. Mol Cancer Ther. 2006;5:995-1006.
[98] Luo Y, Jiang F, Cole TB, Hradil VP, Reuter D, Chakravartty A, et al. A novel multi-targeted tyrosine kinase inhibitor, linifanib (ABT-869), produces functional and structural changes in tumor vasculature in an orthotopic rat glioma model. Cancer Chemother Pharmacol. 2012;69:911-21.
[99] Milne JC, Lambert PD, Schenk S, Carney DP, Smith JJ, Gagne DJ, et al. Small molecule activators of SIRT1 as therapeutics for the treatment of type 2 diabetes. Nature. 2007;450:712-6.

[100] Suzuki K, Hayashi R, Ichikawa T, Imanishi S, Yamada T, Inomata M, et al. SRT1720, a SIRT1 activator, promotes tumor cell migration, and lung metastasis of breast cancer in mice. Oncol Rep. 2012;27:1726-32.
[101] Trudel S, Li ZH, Rauw J, Tiedemann RE, Wen XY, Stewart AK. Preclinical studies of the pan-Bcl inhibitor obatoclax (GX015-070) in multiple myeloma. Blood. 2007;109:5430-8.
[102] Parikh SA, Kantarjian H, Schimmer A, Walsh W, Asatiani E, El-Shami K, et al. Phase II study of obatoclax mesylate (GX15-070), a small-molecule BCL-2 family antagonist, for patients with myelofibrosis. Clin Lymphoma Myeloma Leuk. 2010;10:285-9.
[103] Bae SH, Kim JH, Park TH, Lee K, Lee BI, Jang H. BMS794833 inhibits macrophage efferocytosis by directly binding to MERTK and inhibiting its activity. Exp Mol Med. 2022;54:1450-60.
[104] Yang L, Ding G, Lin H, Cheng H, Kong Y, Wei Y, et al. Transcriptome analysis of medicinal plant Salvia miltiorrhiza and identification of genes related to tanshinone biosynthesis. PLoS One. 2013;8:e80464.
[105] Wang S, Jing H, Yang H, Liu Z, Guo H, Chai L, et al. Tanshinone I selectively suppresses pro-inflammatory genes expression in activated microglia and prevents nigrostriatal dopaminergic neurodegeneration in a mouse model of Parkinson's disease. J Ethnopharmacol. 2015;164:247-55.
[106] Kotian S, Zhang L, Boufraqech M, Gaskins K, Gara SK, Quezado M, et al. Dual Inhibition of HDAC and Tyrosine Kinase Signaling Pathways with CUDC-907 Inhibits Thyroid Cancer Growth and Metastases. Clin Cancer Res. 2017;23:5044-54.
[107] Fu XH, Zhang X, Yang H, Xu XW, Hu ZL, Yan J, et al. CUDC-907 displays potent antitumor activity against human pancreatic adenocarcinoma in vitro and in vivo through inhibition of HDAC6 to downregulate c-Myc expression. Acta Pharmacol Sin. 2019;40:677-88.
[108] Mosqueda J, Olvera-Ramirez A, Aguilar-Tipacamu G, Canto GJ. Current advances in detection and treatment of babesiosis. Curr Med Chem. 2012;19:1504-18.
[109] Wang Y, Wang L, Guan S, Cao W, Wang H, Chen Z, et al. Novel ALK inhibitor AZD3463 inhibits neuroblastoma growth by overcoming crizotinib resistance and inducing apoptosis. Sci Rep. 2016;6:19423.
[110] Asik A, Ay NPO, Bagca BG, Caglar HO, Gunduz C, Avci CB. Combination of Salinomycin and AZD3463 Reveals Synergistic Effect on Reducing the Viability of T98G Glioblastoma Cells. Anticancer Agents Med Chem. 2020;20:2267-73.
[111] Montalvo-Ortiz BL, Castillo-Pichardo L, Hernandez E, Humphries-Bickley T, De la Mota-Peynado A, Cubano LA, et al. Characterization of EHop-016, novel small molecule inhibitor of Rac GTPase. J Biol Chem. 2012;287:13228-38.
[112] Castillo-Pichardo L, Humphries-Bickley T, De La Parra C, Forestier-Roman I, Martinez-Ferrer M, Hernandez E, et al. The Rac Inhibitor EHop-016 Inhibits Mammary Tumor Growth and Metastasis in a Nude Mouse Model. Transl Oncol. 2014;7:546-55.
[113] Martin MW, Newcomb J, Nunes JJ, McGowan DC, Armistead DM, Boucher C, et al. Novel 2-aminopyrimidine carbamates as potent and orally active inhibitors of Lck: synthesis, SAR, and in vivo antiinflammatory activity. J Med Chem. 2006;49:4981-91.
[114] Yamamoto K, Kitayama T, Ishida N, Watanabe T, Tanabe H, Takatani M, et al. Identification and characterization of a potent antibacterial agent, NH125 against drug-resistant bacteria. Biosci Biotechnol Biochem. 2000;64:919-23.
[115] Kim W, Fricke N, Conery AL, Fuchs BB, Rajamuthiah R, Jayamani E, et al. NH125 kills methicillin-resistant Staphylococcus aureus persisters by lipid bilayer disruption. Future Med Chem. 2016;8:257-69.

[116] Blackie JA, Bloomer JC, Brown MJ, Cheng HY, Hammond B, Hickey DM, et al. The identification of clinical candidate SB-480848: a potent inhibitor of lipoprotein-associated phospholipase A2. Bioorg Med Chem Lett. 2003;13:1067-70.
[117] Serruys PW, Garcia-Garcia HM, Buszman P, Erne P, Verheye S, Aschermann M, et al. Effects of the direct lipoprotein-associated phospholipase A(2) inhibitor darapladib on human coronary atherosclerotic plaque. Circulation. 2008;118:1172-82.
